# Integrated metabolomic and transcriptomic analyses of the synergistic effect of polymyxin–rifampicin combination against *Pseudomonas aeruginosa*

**DOI:** 10.1186/s12929-022-00874-3

**Published:** 2022-10-30

**Authors:** Mohd Hafidz Mahamad Maifiah, Yan Zhu, Brian T. Tsuji, Darren J. Creek, Tony Velkov, Jian Li

**Affiliations:** 1 Drug Delivery, Disposition and Dynamics, Monash Institute of Pharmaceutical Sciences, Monash University, Parkville, VIC 3052 Australia; 2 International Institute for Halal Research and Training, International Islamic University Malaysia, 50728 Kuala Lumpur, Malaysia; 3 Infection Program and Department of Microbiology, Monash Biomedicine Discovery Institute, Monash University, Melbourne, VIC 3800 Australia; 4 Department of Pharmacy Practice, School of Pharmacy and Pharmaceutical Sciences, University at Buffalo, Buffalo, NY USA; 5 Department of Biochemistry and Pharmacology, University of Melbourne, Melbourne, VIC 3010 Australia

**Keywords:** Gram-negative bacteria, Antibiotic resistance, Combination therapy, Systems pharmacology, Colistin, Genome-scale metabolic modeling

## Abstract

**Background:**

Understanding the mechanism of antimicrobial action is critical for improving antibiotic therapy. For the first time, we integrated correlative metabolomics and transcriptomics of *Pseudomonas aeruginosa* to elucidate the mechanism of synergistic killing of polymyxin–rifampicin combination.

**Methods:**

Liquid chromatography-mass spectrometry and RNA-seq analyses were conducted to identify the significant changes in the metabolome and transcriptome of *P. aeruginosa* PAO1 after exposure to polymyxin B (1 mg/L) and rifampicin (2 mg/L) alone, or in combination over 24 h. A genome-scale metabolic network was employed for integrative analysis.

**Results:**

In the first 4-h treatment, polymyxin B monotherapy induced significant lipid perturbations, predominantly to fatty acids and glycerophospholipids, indicating a substantial disorganization of the bacterial outer membrane. Expression of ParRS, a two-component regulatory system involved in polymyxin resistance, was increased by polymyxin B alone. Rifampicin alone caused marginal metabolic perturbations but significantly affected gene expression at 24 h. The combination decreased the gene expression of quorum sensing regulated virulence factors at 1 h (e.g. key genes involved in phenazine biosynthesis, secretion system and biofilm formation); and increased the expression of peptidoglycan biosynthesis genes at 4 h. Notably, the combination caused substantial accumulation of nucleotides and amino acids that last at least 4 h, indicating that bacterial cells were in a state of metabolic arrest.

**Conclusion:**

This study underscores the substantial potential of integrative systems pharmacology to determine mechanisms of synergistic bacterial killing by antibiotic combinations, which will help optimize their use in patients.

**Graphical Abstract:**

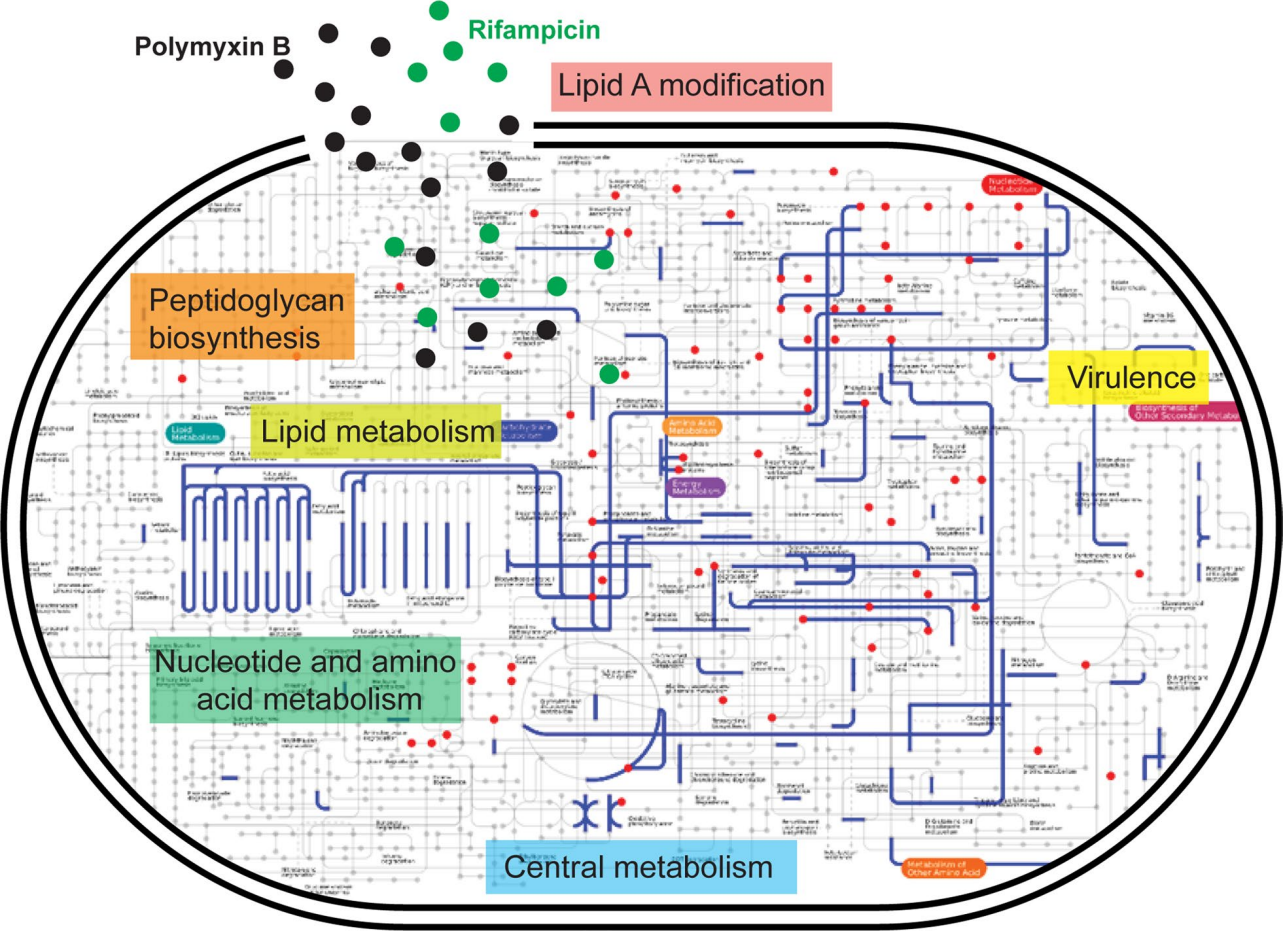

**Supplementary Information:**

The online version contains supplementary material available at 10.1186/s12929-022-00874-3.

## Background

*Pseudomonas aeruginosa* is a major opportunistic Gram-negative pathogen with a large genome (~ 6 Mb) encoding significant metabolic flexibility and many virulence factors [1, 2]. This organism is intrinsically resistant to many antibiotics and has a high propensity to develop resistance to all currently available anti-pseudomonals when used as monotherapy [3]. Multidrug-resistant (MDR) *P. aeruginosa* is a common cause of severe infections in healthcare settings and immunocompromised patients, and are associated with high rates of morbidity and mortality [4]. Worryingly, the prevalence of MDR strains is increasing globally [5–8], with ~ 13% of severe healthcare-associated infections in the United States attributable to MDR *P. aeruginosa* [9]. Given the threat posed by this difficult-to-treat organism, the World Health Organization (WHO) declared carbapenem-resistant *P. aeruginosa* one of the three Priority 1 (Critical) pathogens urgently requiring novel antibiotic treatments while the Centers for Disease Control and Prevention (CDC) designated MDR *P. aeruginosa* as a Serious threat [9, 10].

The combination of the rapid emergence of MDR Gram-negative pathogens over the last few decades and lack of development of new antimicrobials [11, 12] has forced clinicians to re-examine the ‘old’ polymyxin class of antibiotics [13, 14]. Polymyxins are non-ribosomal poly-cationic cyclic lipopeptides with only polymyxin B and E (the latter known as colistin) available for use in the clinic [15]. Polymyxins were rarely used during the 1970s and 1990s due to concerns over nephrotoxicity which may occur in up to 60% of patients receiving intravenous polymyxin therapy [16]. However, with ever increasing resistance to the other antibiotics, polymyxin use has rapidly increased over the last two decades given they retain activity against problematic Gram-negative bacteria, including *P. aeruginosa* [17, 18]. Indeed, many MDR Gram-negative pathogens are only susceptible to the polymyxins [19].

While the precise mechanism(s) by which polymyxins ultimately kill bacteria remains unknown, initial electrostatic interactions between the cationic polymyxins and anionic phosphate groups of the lipid A moiety of lipopolysaccharide (LPS) in the Gram-negative outer membrane are a requirement for activity [20]. However, reports of polymyxin-resistant *P. aeruginosa* and other Gram-negative pathogens are increasing [21], with most reported resistance mechanisms involving lipid A modifications [22–25]. Pharmacokinetic/pharmacodynamic (PK/PD) studies have shown the potential for the rapid emergence of resistance with polymyxin monotherapy, including treatments when polymyxins are used at concentrations exceeding those achieved in patients [26]. Therefore, polymyxin combination therapy has been suggested to enhance efficacy and minimize toxicity and the emergence of resistance [27, 28]. Polymyxins in combination with rifampicin are synergistic against MDR Gram-negative pathogens, including *P. aeruginosa* [29–33]. While it is known that disruption of the bacterial outer membrane by polymyxins facilitates penetration of rifampicin [31, 34–36], subsequent gene expression and cellular metabolic events which result in enhanced bacterial killing are unclear.

The present study is the first to integrate metabolomics and transcriptomics to investigate enhanced killing of *P. aeruginosa* by a polymyxin–rifampicin combination. Significant perturbations of bacterial metabolic and regulatory networks were identified with the combination, in particular changes in cross-membrane transport, lipid and carbohydrate metabolism, quorum sensing, and virulence. Our study highlights the potential of polymyxin combinations for the treatment of MDR *P. aeruginosa* infections.

## Materials and methods

### Strain, antibiotics and reagents

*Pseudomonas aeruginosa* PAO1 (hereafter PAO1) was from the American Type Culture Collection (ATCC). The minimum inhibitory concentrations (MICs) of PAO1 to polymyxin B (0.5 mg/L) and rifampicin (32 mg/L) were determined via broth microdilution in cation-adjusted Mueller–Hinton broth (CAMHB; Oxoid, Australia; 20–25 mg/L Ca^2+^ and 10–12.5 mg/L Mg^2+^) according to the Clinical and Laboratory Standards Institute guidelines [37]. Prior to experiments, solutions of polymyxin B (sulfate; Sigma-Aldrich, Castle Hill, NSW, Australia; batch number: BCBD1065V) were prepared using Milli-Q water (Millipore, North Ryde, New South Wales, Australia) and rifampicin (Sigma-Aldrich; batch number: 011M1159V) using dimethyl sulfoxide (DMSO; Sigma-Aldrich). Stock solutions were sterilized by filtration with a 0.22-µm pore size Millex GP filter (Millipore) prior to dilution in sterilized Milli-Q water.

### Bacterial culture preparation and time-kill kinetics

PAO1 was subcultured on nutrient agar from frozen stock (− 80 °C) and incubated for 16–18 h at 37 °C. Single colonies were subsequently inoculated into 15 mL of CAMHB and incubated for 16–18 h at 37 °C with shaking at 150 rpm. The overnight cultures were diluted (1:100) into four different reservoirs of 200 mL fresh CAMHB (one for each of three treatment regimens plus the control). To prevent excessive bacterial killing and obtain enough cells for the metabolomic and transcriptomic experiments, bacterial cultures were grown to an optical density at 600 nm (OD_600_) of ~ 0.5 (~ 10^8^ CFU/mL) prior to addition of antibiotic(s). Considering the inoculum effect of polymyxin killing [38, 39], a preliminary time-kill study was conducted with an initial inoculum of ~ 1 × 10^8^ CFU/mL over 24 h. Our preliminary results demonstrated synergistic activity at least over the first 4 h by the combination of polymyxin B (2 mg/L) and rifampicin (4 mg/L) (1.75 × 10^5^, 3.42 × 10^5^, 9.67 × 10^6^ CFU/mL at 0.5, 1 and 4 h, respectively), with > 2 log_10_ CFU/mL additional killing over polymyxin B alone (2.62 × 10^7^, 1.35 × 10^8^, 1.11 × 10^9^ CFU/mL at 0.5, 1 and 4 h, respectively); no killing was observed at any time points by rifampicin alone. Therefore, in the metabolomic and transcriptomic studies bacteria were treated with either (i) polymyxin B (1 mg/L), (ii) rifampicin (2 mg/L), and (iii) the combination of polymyxin B (1 mg/L) and rifampicin (2 mg/L); these concentrations of polymyxin B and rifampicin were chosen based on clinical relevance [40, 41] and to obtain sufficient bacterial cells for metabolomic and transcriptomic studies [42]. Untreated bacterial cultures served as controls. The metabolomic and transcriptomic studies were conducted with four and three biological replicates, respectively, on different days.

### RNA extraction and analysis of RNA-seq data

For RNA extraction, samples of bacterial cultures (1.5 mL) were collected immediately before antibiotic treatment (i.e., 0 h) and at 1 h and 24 h after treatment. All samples were normalized to an optical density (OD_600_) of ~ 0.5 (~ 10^8^ CFU/mL) with fresh CAMHB. RNA was extracted according to the RNeasy Mini Kit manufacturer’s protocol (Qiagen) [43] and its quality and quantity were checked using Nanodrop (Thermo Fisher Scientific). All samples (*n* = 27) were subjected to RNA-seq (100-bp single-end) using Illumina HiSeq 1500 platform at Hudson Medical Research Institute (Clayton, Victoria, Australia). The reads were aligned to the PAO1 genome obtained from the *Pseudomonas* Genome Database [44] using SubRead [45] with default settings. The counts of mapped reads were summarized by FeatureCounts [45]. Overall, 97,676,777 raw reads were obtained for 1 and 24 h (*n* = 3), with 63.7–97.1% of reads successfully aligned to the coding regions of *P. aeruginosa* PAO1 genome [1]. Differential gene expression was identified using Degust (www.vicbioinformatics.com/degust), a graphic interface of Voom and Limma packages [46]. The statistical significance of differential gene expression (DEG) was determined with Benjamini–Hochberg adjustment to control the false discovery rate (FDR). DEG was defined with a combination of fold change (FC) > 2 (i.e. > 1.0 and < − 1.0 log_2_FC) and FDR ≤ 0.05. Gene ontology enrichment analysis of DEGs of the polymyxin B and rifampicin combination was performed using Reduce and Visualize Gene ontology (REVIGO) [47].

### Preparation of cellular metabolite extracts

Cellular metabolites were extracted by a previously optimized method with slight modifications [48]. Samples for metabolite extraction and viable counting were collected immediately before antibiotic treatment (i.e., 0 h) and at 0.25, 1, 4 and 24 h after treatment. For the fingerprint samples (i.e., intracellular metabolites), 20 mL of the bacterial culture was collected and immediately transferred onto ice. The samples were then quenched in a dry ice/ethanol bath and preserved on ice for all the following steps. Samples were normalized to an optical density (OD_600_) of ~ 0.5 (~ 10^8^ CFU/mL) with fresh CAMHB and 10 mL transferred into new 15 mL falcon tubes (Thermo Fisher, Australia) for immediate metabolite extraction. Samples were then centrifuged for 10 min at 3220×*g* at 4 °C. Cell pellets were thrice washed with 0.9% NaCl (4 °C) and centrifuged at 3220×*g* for 3 min at 4 °C. Cell pellets were then resuspended in 250 µL of chloroform:methanol:water (CMW; 1:3:1, v/v; − 80 °C) containing generic and physiologically diverse internal standards (CHAPS, CAPS, PIPES and TRIS; all 1 µM) for cellular metabolite extraction. Samples were immediately frozen in liquid nitrogen and allowed to thaw on ice. This freeze–thaw process was repeated three times after which the samples were vortexed to lyse the cells and release cellular metabolites. The extracted samples were centrifuged at 3220×*g* for 10 min at 4 °C and the supernatant collected and further centrifuged at 14,000×*g* for 10 min at 4 °C to remove cell debris. The final particle-free supernatant samples (200 μL) were transferred to injector vials for LC–MS analysis. For footprint samples, aliquots of the supernatant were rapidly filtered through a 0.22-µm membrane filter; 10 μL of the supernatant was mixed with 250 μL of CMW (1:3:1, v/v) and centrifuged at 14,000×*g* for 10 min at 4 °C to collect particle-free supernatant for LC–MS analysis.

### LC–MS analysis of metabolites

LC–MS analyses were performed on a Q-Exactive Orbitrap mass spectrometer (Thermo Fisher) coupled to a Dionex high-performance liquid chromatograph (U3000 RSLC HPLC, Thermo Fisher) with a ZIC-pHILIC column (5 μm, polymeric, 150 × 4.6 mm; SeQuant, Merck). The MS system was operated at 35,000 resolution in both positive and negative electro-spray ionization (ESI) mode (rapid switching) with a detection range of 85 to 1275 m*/z*. The LC solvent consisted of 20 mM ammonium carbonate (A) and acetonitrile (B) with a multi-step gradient system from 80% B to 50% B over 15 min, then to 5% B at 18 min, followed by a wash with 5% B for 3 min and re-equilibration for 8 min with 80% B, with a flow rate of 0.3 mL/min [49]. The injection sample volume was 10 μL and the run time was 32 min. All samples were analyzed in the same run and the chromatographic peaks, signal reproducibility and analyte stability monitored by assessment of pooled quality control samples (10 μL aliquot of each sample, including both footprints and fingerprints) analyzed periodically throughout the batch. Mixtures of pure standards containing over 200 metabolites were included and analyzed within the batch to assist metabolite identification.

### Data processing, bioinformatics and statistical analyses

Metabolomic data analyses were performed as previously described [48] using IDEOM (http://mzmatch.sourceforge.net/ideom.php) [50]. The quantification of each metabolite was based on the chromatogram raw peak height and univariate and multivariate analyses conducted with MetaboAnalyst 3.0 [51]. Prior to analysis, relative peak intensity data were normalized by the median, log transformed and scaled (by auto scale function) to reduce variance between the samples. Unsupervised principal component analysis (PCA) was conducted with global metabolic profiles at each time point. Significantly changed metabolites of treated samples relative to untreated control samples at each time point were identified by One-way Analysis of Variance (ANOVA, FDR ≤ 0.05) followed by post-hoc analysis using Tukey’s Honestly Significant Difference (Tukey’s HSD). Metabolites with > 1.0 and < − 1.0 log_2_FC were further analyzed and subjected to metabolic pathway analysis.

### Reconstruction of genome-scale metabolic network (GSMN) for *P. aeruginosa* PAO1

We previously constructed a GSMN *i*PAO1 for *P. aeruginosa* PAO1 containing a total of 4265 reactions, 3022 metabolites, 1458 genes and 110 metabolic pathways [52]. The metabolomic and transcriptomic data were casted, mapped to and visualized on the metabolic network of *i*PAO1 using VANTED [53]. The significantly changed genes and metabolites of the polymyxin B/rifampicin combination at 1 h and 24 h were mapped using iPath (http://pathways.embl.de/index.html) [54] to demonstrate the general overview of the metabolic pathway perturbations using KEGG.

## Results

### Significantly perturbed metabolome induced by polymyxin B and rifampicin alone and in combination

**Fig. 1:**
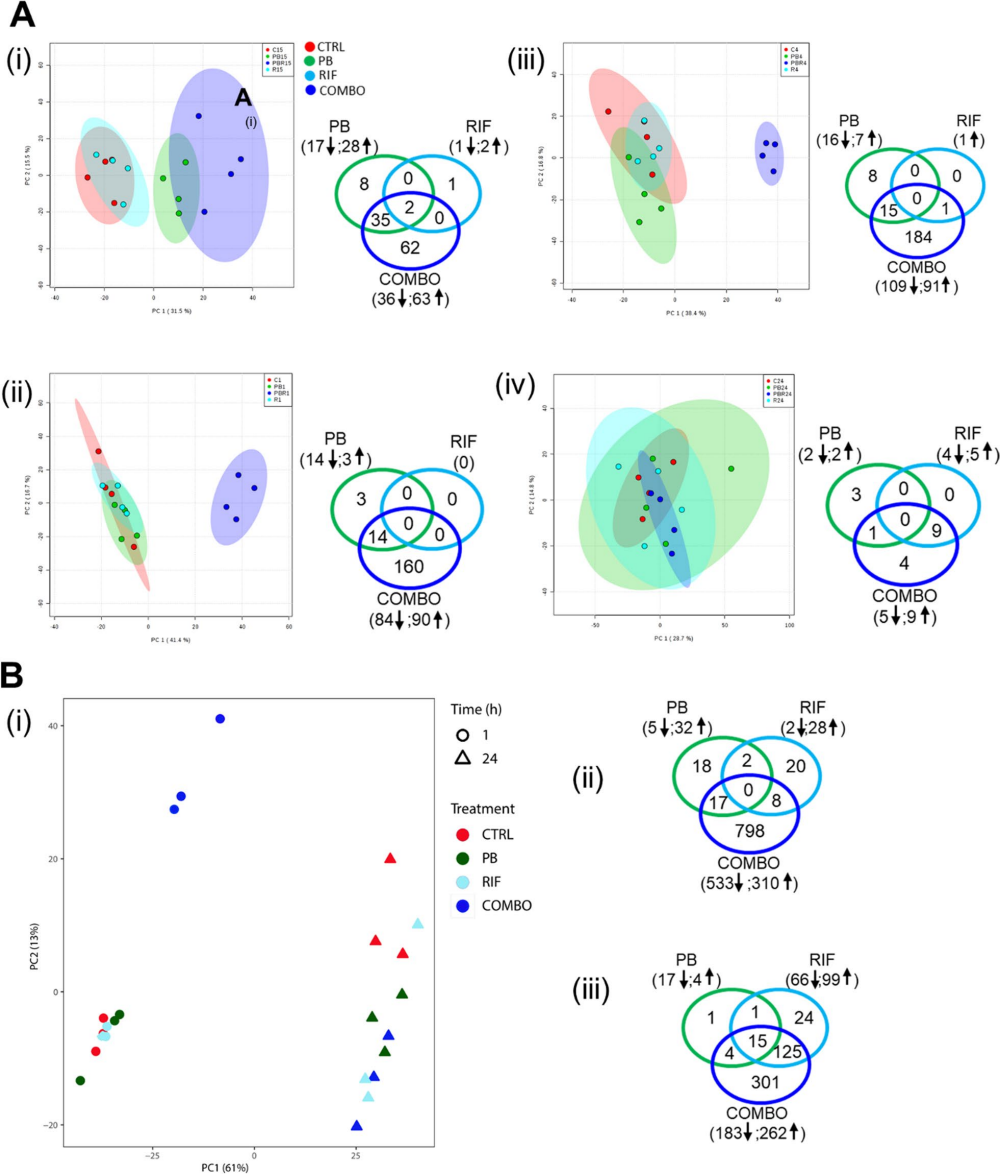
**A** PCA plots (left) and Venn diagrams (right) of global metabolic changes in response to no treatment (CTRL) or treatment with polymyxin B (PB) or rifampicin (RIF) alone, and in combination (COMBO), at (i) 0.25 h, (ii) 1 h, (iii) 4 h and (iv) 24 h. **B** (i) PCA plot and Venn diagrams of gene expression changes at (ii) 1 h and (iii) 24 h. The numbers in brackets represent the metabolites or genes that were significantly changed (up- and down-regulated). Significantly changed metabolites and genes were identified with > 1.0 and < − 1.0 log_2_FC and FDR ≤ 0.05

Polymyxin B was employed as the representative of polymyxins in this study. Clinically relevant concentrations of polymyxin B (1 mg/L) and rifampicin (2 mg/L) were investigated [40, 41]; 0.25 h, 1 h and 4 h were examined for the initial antibacterial effect while 24 h was for persistent effect and potential emergence of resistance. Overall, 2520 metabolites in PAO1 were putatively identified. The Principal Component Analysis (PCA) results showed that polymyxin B alone slightly overlapped with the combination at 0.25 h (Fig. 1A(i)). Whereas from 1 h onwards polymyxin B and rifampicin monotherapies were grouped together with the untreated control with minor metabolic changes observed (Fig. 1A(ii–iv)). With rifampicin, the very minimal metabolites changed (Additional file 1: Tables S1–S4) was consistent with the complete lack of bacterial killing observed when used as monotherapy (Additional file 2: Fig. S1). The combination at 0.25 h was distinct from the untreated control with 99 significantly changed metabolites and 37 of these 99 metabolites were significantly perturbed by polymyxin B monotherapy (Fig. 1A(i), Additional file 1: Table S1). Our results showed that at early stage the combination was mostly driven by polymyxin B. At 1 and 4 h, 174 and 200 metabolites were significantly changed by the combination, respectively; while only 14 and 15 metabolites were perturbed by polymyxin B alone correspondingly. However, at 24 h only 14 significantly altered metabolites were observed by the combination, including 9 metabolites significantly changed by rifampicin alone and only one metabolite by polymyxin B alone (Fig. 1A(iv), Additional file 1: Table S4).

### Gene expression changes following treatments of polymyxin B and rifampicin alone and in combination

PCA results show drastic gene expression variations induced by the treatment duration (PC1) and treatment groups (PC2). Of particular interest is that the combination exhibits a distinct gene expression pattern compared to the untreated control and each monotherapy (Fig. 1B(i)). In contrast to each antibiotic alone, the polymyxin B/rifampicin combination caused substantial perturbations in gene expression, evident by 823 and 445 differentially expressed genes at 1 and 24 h, respectively; this is consistent with their synergistic activity against *P. aeruginosa* PAO1 (Fig. 1B(ii, iii), Additional file 1: Tables S7 and S10). Approximately 97% (798/823) and 68% (301/445) of the genes were exclusively expressed by the combination only at 1 and 24 h, respectively. Interestingly, at 24 h there were 15 common differentially expressed genes induced by the monotherapies and the combination (Table 1). Further gene ontology (GO) enrichment shows that the polymyxin B/rifampicin combination significantly perturbed various biological processes at 1 and 24 h, in particular transmembrane transport, lipid and carbohydrate metabolism, virulence, and phosphorylation (Additional file 2: Fig. S2).

**Table 1:** Common differentially expressed genes (> 1.0 and < − 1.0 log_2_FC and FDR ≤ 0.05) induced by polymyxin B (PB) alone, rifampicin (RIF) alone and the combination (COMBO) at 24 h

Locus tag (gene name)	Product description	Expression ratio (log_2_)		
		PB	RIF	COMBO
PA1483 (*CycH*)	Cytochrome c-type biogenesis protein CycH	− 1.76	− 1.87	− 2.01
PA2006	Major facilitator superfamily transporter	2.52	3.19	3.29
PA2006 (*NuoG*)	NADH-quinone oxidoreductase subunit G	− 1.33	− 1.63	− 1.88
PA2993	Hypothetical protein	− 1.66	− 1.94	− 1.99
PA3495 (*Nth*)	Endonuclease III	− 1.63	− 2.02	− 1.66
PA3559	Nucleotide sugar dehydrogenase	− 2.46	− 3.04	− 3.42
PA3650 (*Dxr*)	1-Deoxy-d-xylulose 5-phosphate reductoisomerase	− 1.78	− 2.78	− 3.19
PA3800	OM protein assembly factor BamB	− 1.67	− 1.91	− 1.81
PA3801	Hypothetical protein	− 1.78	− 2.27	− 2.21
PA4251 (*RplE*)	50S ribosomal protein L5	− 2.53	− 3.01	− 2.88
PA4252 (*RplX*)	50S ribosomal protein L24	− 2.60	− 3.08	− 3.22
PA4253 (*RplN*)	50S ribosomal protein L14	− 2.25	− 2.94	− 3.16
PA5001	Hypothetical protein	− 1.43	− 1.96	− 1.98
PA5007	Hypothetical protein	− 2.09	− 2.16	− 2.14
PA5365 (*PhoU*)	Phosphate uptake regulatory protein PhoU	− 1.66	− 2.25	− 2.07

### Integration of metabolomic and transcriptomic data

To elucidate the dynamic changes of gene expression and cellular metabolism in response to the treatments, we mapped the correlative transcriptomic and metabolomic data to the metabolic network (*i*PAO1) of *P. aeruginosa* PAO1 and analyzed the underlying mechanism in a pathway-specific manner (Additional file 2: Fig. S3). The following sections highlight the significant biochemical pathway changes induced by mono- and combination therapies.

### Polymyxin-induced cell envelope changes and lipid A modification

**Fig. 2:**
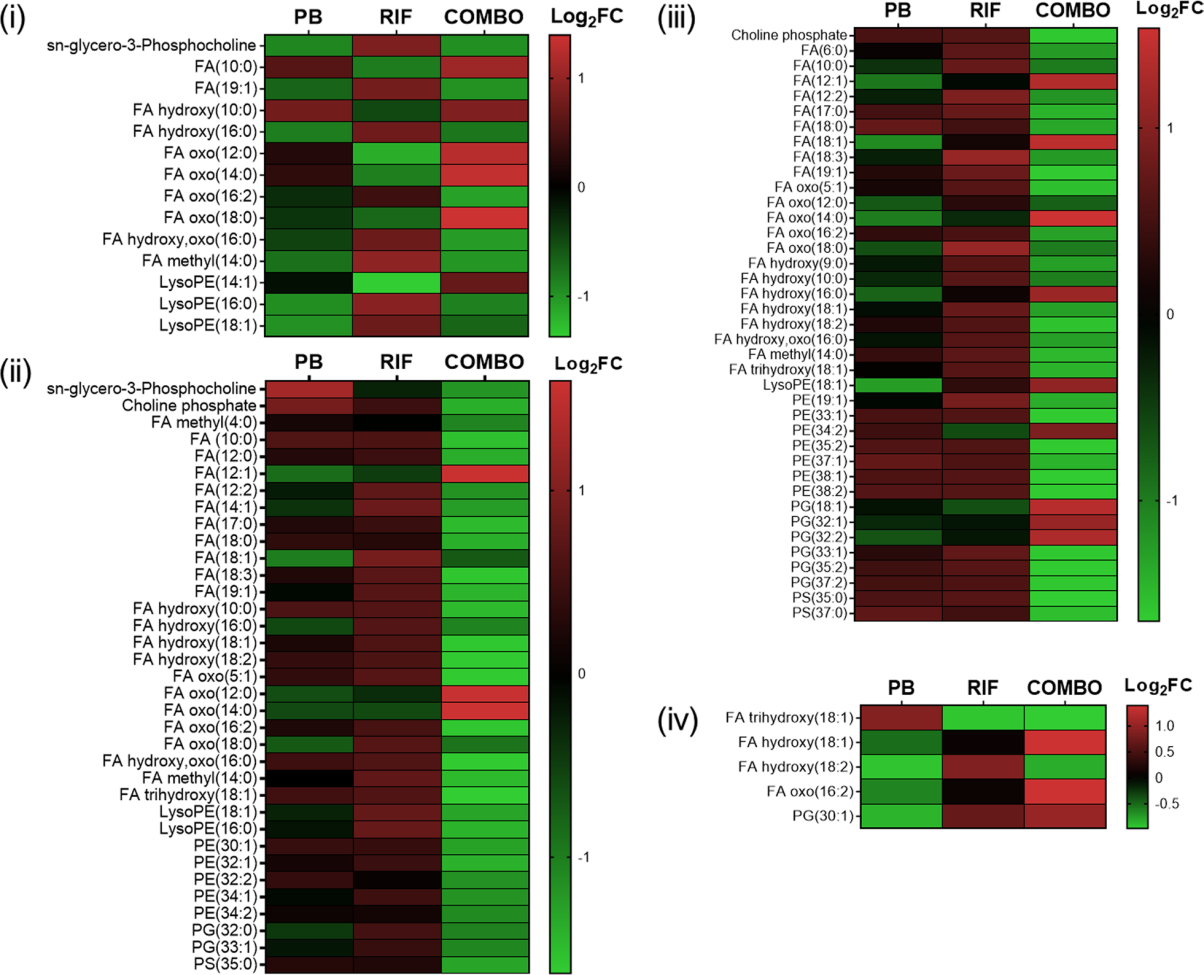
Perturbations of membrane-related lipids. Heatmap profiles of the significantly changed lipids at **i** 0.25 h, **ii** 1 h, **iii** 4 h and **iv** 24 h. Polymyxin B alone and the polymyxin B/rifampicin combination predominantly altered the fatty acids and glycerophospholipids of PAO1 at 0.25, 1 and 4 h. Few fatty acids were perturbed by either monotherapy or the combination at 24 h. Lipids were putatively annotated with reference to accurate mass. *PB* polymyxin B, *RIF* rifampicin, *COMBO* polymyxin B/rifampicin combination. Significantly changed metabolites were identified with > 1.0 and < − 1.0 log_2_FC and FDR ≤ 0.05

Across the first 4 h, polymyxin B monotherapy and the polymyxin B/rifampicin combination significantly perturbed (> 1.0 log_2_ fold change [FC], false discovery rate [FDR] ≤ 0.05) membrane-associated lipids, particularly fatty acids and glycerophospholipids, with the effect of the combination at 1 and 4 h greater than that of polymyxin B monotherapy (Fig. 2). At these time points, phosphatidylethanolamine (PE) and phosphatidylglycerol (PG) species with long fatty acyl chains were markedly altered by the combination, compared to polymyxin B monotherapy. Consistently, the gene expression of fatty acid biosynthesis (PA5524, PA0098, *fabl*, *fabH2*, *atoB*) and phospholipase (*pldA*) was altered by the combination at 1 h (Additional file 1: Table S7). At late stage (24 h), the abundance of several fatty acids was significantly perturbed by rifampicin alone and the combination (Fig. 2); while phosphatidate cytidylyltransferase gene *cdsA* was downregulated by 1.7-fold and 2.3-fold, respectively (Additional file 1: Tables S9 and S10).

**Fig. 3:**
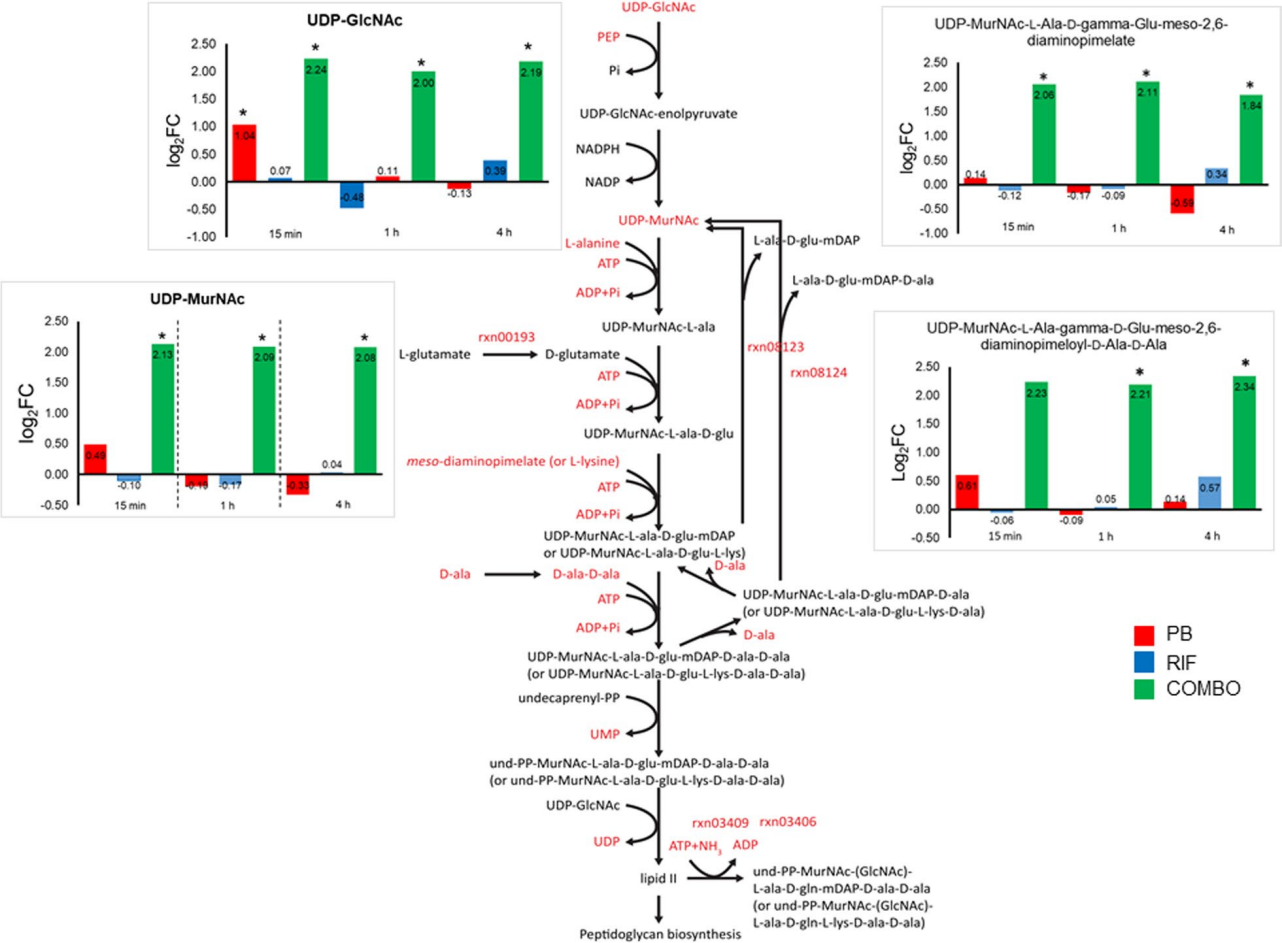
Perturbations of the peptidoglycan biosynthesis pathway. The polymyxin B/rifampicin combination significantly upregulated intracellular metabolites and differentially expressed genes (DEGs) associated with peptidoglycan biosynthesis over the first 4 h of treatment. *PB* polymyxin B, *RIF* rifampicin, *COMBO* polymyxin B/rifampicin combination. Red and * indicates the significantly changed metabolites and DEGs that were identified with > 1.0 and < − 1.0 log_2_FC and FDR ≤ 0.05

**Fig. 4:**
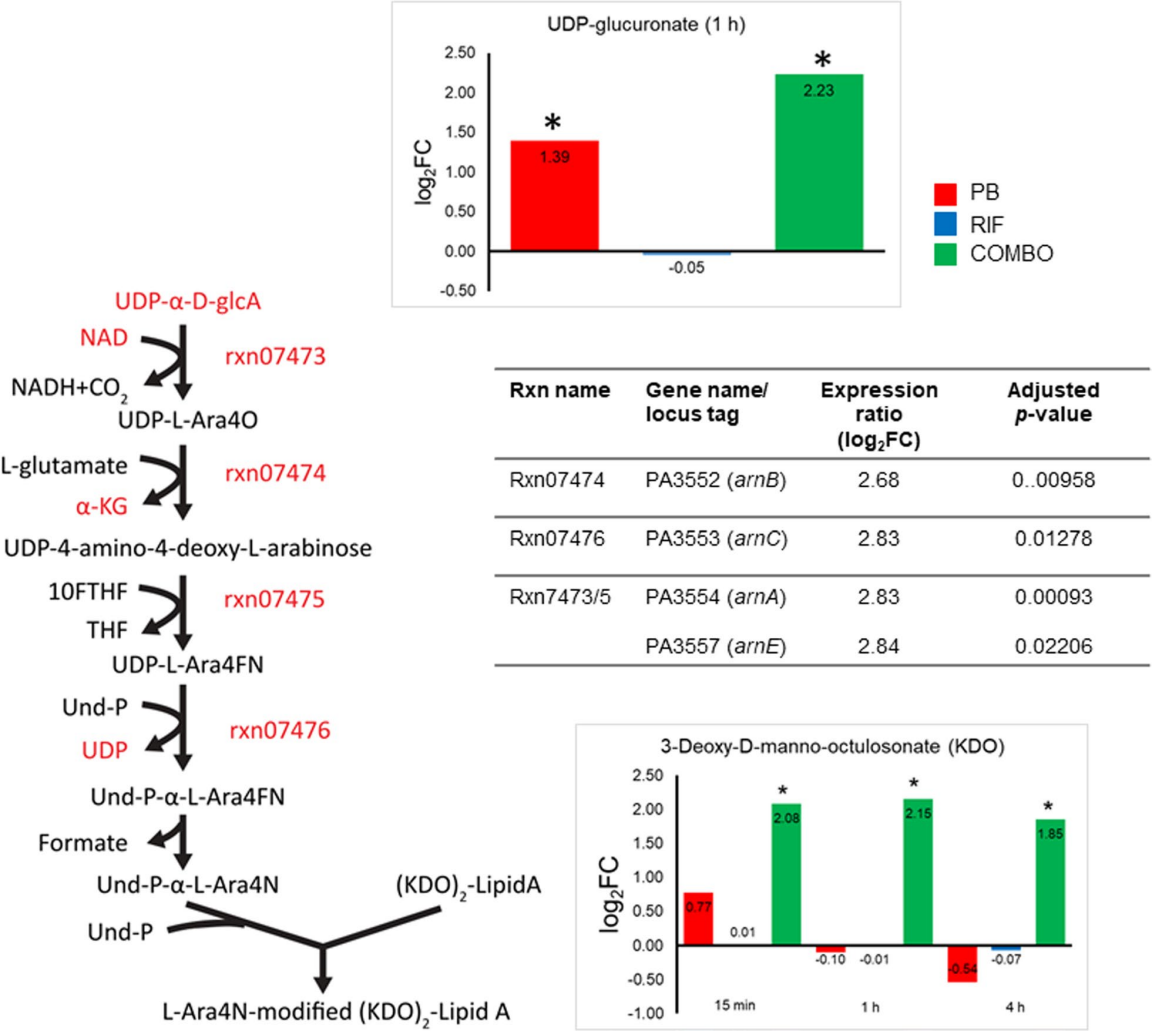
Perturbations of the LPS modification pathway at 1 h. Polymyxin B alone significantly upregulated *arnA* (rxn07473, rxn07475), *arnB* (rxn07474), *arnC* (rxn07476) and UDP-glucoronate causing the addition of 4-aminoarabinose to the lipid A of LPS. PB, polymyxin B; RIF, rifampicin; COMBO, polymyxin B/rifampicin combination. Red and * indicate the significantly changed metabolites and differentially expressed genes that were identified with > 1.0 and < − 1.0 log_2_FC and FDR ≤ 0.05

Significant changes in metabolites of LPS and cell wall biosynthesis were observed only with the combination and only across the first 4 h of treatment. The abundance of several key intermediate metabolites of cell wall biosynthesis, namely UDP-*N*-acetylglucosamine (UDP-GlcNAc), UDP-*N*-acetylmuramate (UDP-MurNAc), UDP-MurNAc-l-Ala-gamma-d-Glu-*meso*-2,6-diaminopimelate and UDP-MurNAc-l-Ala-gamma-d-Glu-*meso*-2,6-diaminopimeloyl-d-Ala-d-Ala, were all significantly elevated by at least 1.8 fold at 0.25, 1 and 4 h in response to the combination treatment (Fig. 3). Likewise, an intermediate metabolite of LPS synthesis (3-deoxy-d-*manno*-octulosonate, KDO) and a precursor metabolite for peptidoglycan and LPS synthesis (d-sedoheptulose 7-phosphate) were both significantly increased by the combination at these time points (Fig. 4). The transcriptomic result also showed a significant increase in the expression of a gene involved with peptidoglycan biosynthesis (*ampDh3*; > 1.0 log_2_FC, FDR ≤ 0.05) at 1 h with combination therapy (Additional file 1: Table S7). Two genes associated with biosynthesis of (KDO)_2_-lipid A (*lpxB*) and peptidoglycan (*murL*) were significantly downregulated (< − 1.0 log_2_FC, FDR ≤ 0.05) at 24 h by rifampicin alone and the combination (Additional file 2: Table S12). Lipid A modification genes *arnA*, *arnB*, *arnC*, *arnE* (Fig. 4) and *pagL* were significantly upregulated only in response to polymyxin B monotherapy, particularly at 1 h. Upregulation of these genes is in line with the significant increase (> 1.4 log_2_FC, FDR ≤ 0.05) in UDP-glucoronate, a key precursor for LPS biosynthesis, observed with both polymyxin B monotherapy and the combination (Fig. 4).

### The polymyxin B/rifampicin combination significantly perturbed nucleotide and amino acid metabolism

**Fig. 5:**
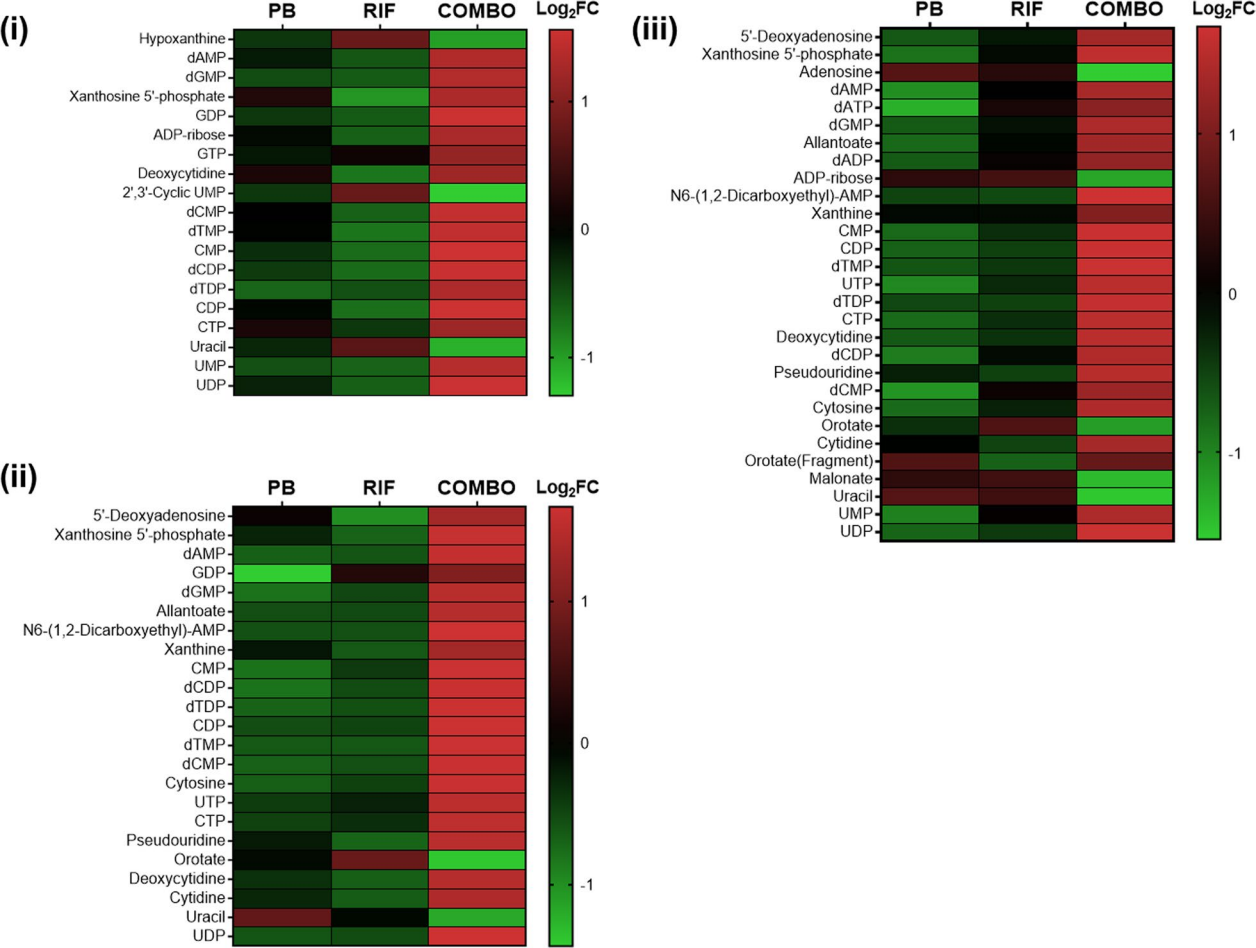
Perturbations of purine and pyrimidine nucleotides. The polymyxin B/rifampicin combination induced significant increases in the levels of a large number of nucleotides at **i** 0.25 h, **ii** 1 h and **iii** 4 h. There were no significant changes in nucleotide levels at 24 h following treatment with either monotherapy or the combination. No treatment (CTRL); *PB* polymyxin B, *RIF* rifampicin, *COMBO* polymyxin B/rifampicin combination. Significantly changed metabolites were identified with > 1.0 and < − 1.0 log_2_FC and FDR ≤ 0.05

Over the first 4 h, neither rifampicin nor polymyxin B monotherapy induced significant changes in nucleotide metabolism, whereas the levels of purine and pyrimidine nucleotides were significantly altered in response to the combination treatment with a majority of nucleotides increased (> 1.0 log_2_FC, FDR ≤ 0.05; Fig. 5). At 1 h, both transcriptomic and metabolomic data showed significant changes in amino acid metabolism following combination therapy only (Table 2). Specifically, the combination significantly altered the biosynthesis of alanine, serine, glycine and phenylalanine, as well as increasing the degradation of valine, leucine, histidine and tyrosine. Notably, at 1 h there was an increase in the relative abundance of 3-methylbutanoyl-CoA (> 2.0 log_2_FC, FDR ≤ 0.05), a metabolite associated with leucine degradation; and a reduction in the expression of genes (*liuA*, *liuB*, *liuE*; < − 2.0 log_2_FC, FDR ≤ 0.05) associated with the breakdown of this metabolite. Similarly, the relative abundance of tyrosine was significantly increased (> 2.0 log_2_FC, FDR ≤ 0.05) while the expression of genes associated with its degradation, namely *phhC*, *hpd*, *hmgA*, *maiA*, and *fahA*, was significantly decreased (< − 1.0 log_2_FC, FDR ≤ 0.05). At 24 h, while genes associated with leucine and tyrosine degradation were significantly upregulated by both rifampicin monotherapy and the combination, no changes in associated metabolites were detected at this timepoint (Table 3).

**Table 2:** Significantly changed metabolites and differentially expressed genes (DEGs; > 1.0 and < − 1.0 log_2_FC and FDR ≤ 0.05) associated with amino acid metabolism induced by the polymyxin B/rifampicin combination at 1 h

Metabolism	Metabolite	Log_2_FC	DEG	Log_2_FC
Alanine biosynthesis	Pyruvate	− 2.07	*iscS*	− 1.17
	l-Alanine	− 1.74	*dadX*	1.93
	Valine	2.07		
Serine and glycine biosynthesis	3-Phospho-d-glycerate	2.15	*glyA2*	1.34
	*O*-Phospho-l-serine	2.03		
	Serine	− 2.01		
Phenylalanine biosynthesis	Phenylpyruvate	− 1.96	*phhC*	− 1.02
Valine degradation	Valine	2.07	*bkdA2*	− 2.72
			PA0744	− 2.47
			PA0743	− 1.90
Leucine degradation	3-Methylbutanoyl-CoA	2.20	*liuA*	− 2.68
			*liuB*	− 2.64
			*liuE*	− 3.51
Histidine degradation	Urocanate	1.77	PA5106	1.12
	*N*-Formimino-l-glutamate	1.54		
Tyrosine degradation	l-Tyrosine	2.01	*phhC*	− 1.02
			*hpd*	− 3.37
			*hmgA*	− 2.75
			*maiA*	− 2.93
			*fahA*	− 2.82

No significantly changed metabolites and DEGs at this time were detected with either monotherapy

**Table 3:** Differentially expressed genes (> 1.0 log_2_FC and FDR ≤ 0.05) at 24 h associated with amino acid metabolism induced by rifampicin (RIF) monotherapy and the polymyxin B/rifampicin combination (COMBO)

Metabolism	RIF	Log_2_FC	COMBO	Log_2_FC
Leucine degradation	*liuA*	2.28	*liuA*	1.76
	*liuB*	2.12	*liuB*	1.76
Tyrosine degradation	*hpd*	3.38	*phhC*	1.79
	*maiA*	2.49	*hpd*	3.26
	*fahA*	3.33	*maiA*	2.98
			*fahA*	3.99

### The polymyxin B/rifampicin combination significantly perturbed central carbon metabolism

**Fig. 6:**
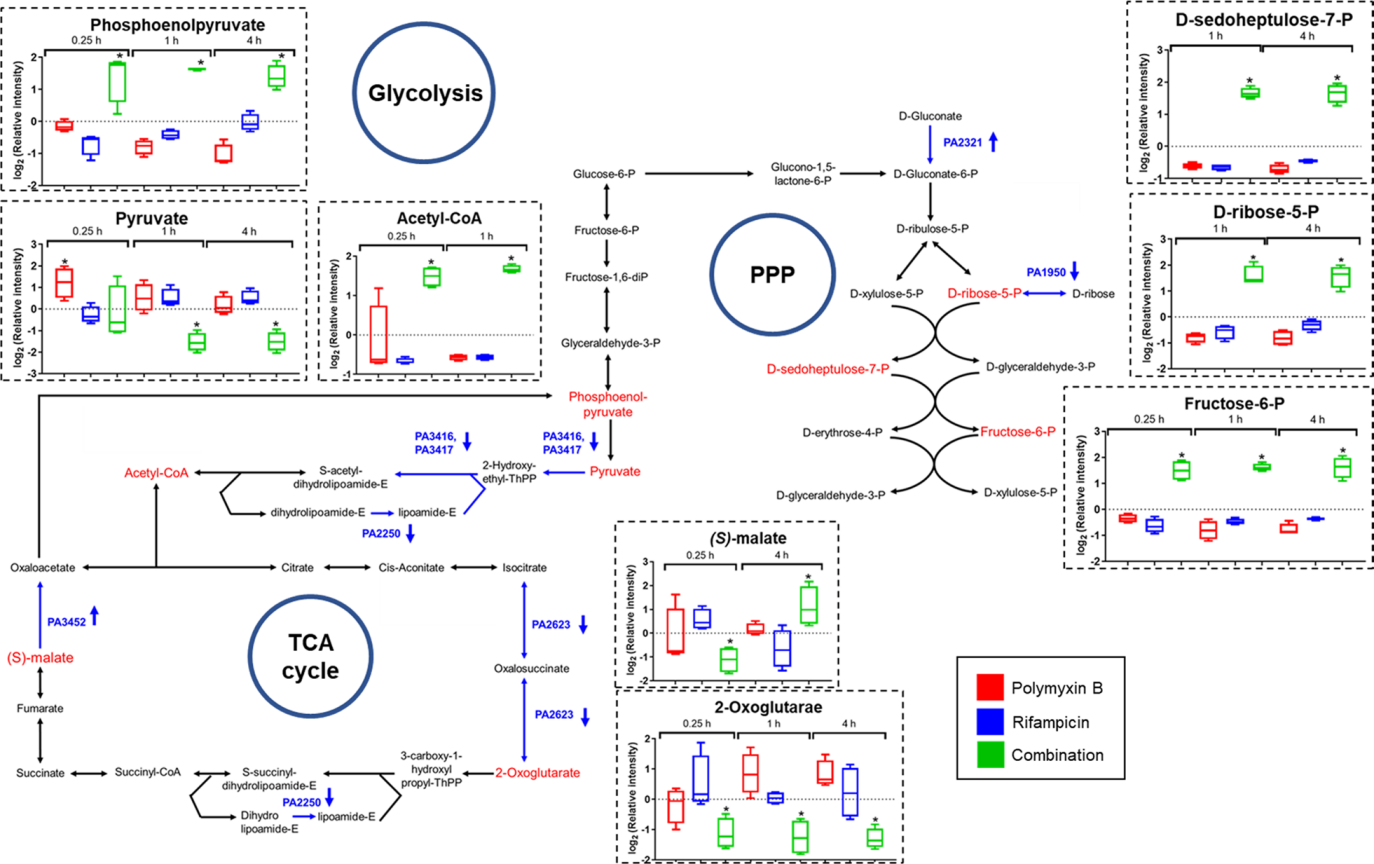
Perturbations of central carbon metabolism by the polymyxin B/rifampicin combination. The combination significantly altered metabolite levels and gene expression associated with the TCA cycle, glycolysis, and the pentose phosphate pathway (PPP), with significantly changed metabolites at 0.25, 1, and 4 h shown in red. Differentially expressed genes (DEGs) at 1 h (either upregulated or downregulated as indicated with arrows) are shown in blue. Box plots show the upper and lower quartiles (top and bottom of box), median (line within box), and the spread of data that are not outliers (whiskers). No significantly altered metabolites or DEGs were observed at 24 h with either monotherapy or the combination. Significantly changed metabolites and DEGs were identified with > 1.0 and < − 1.0 log_2_FC and FDR ≤ 0.05. The figure is modified from the KEGG with reference to *P. aeruginosa* PAO1

Metabolomic (at 0.25, 1 and 4 h) and transcriptomic (at 1 h) effects of the polymyxin B/rifampicin combination on glycolysis, the tricarboxylic acid (TCA) cycle and the pentose phosphate pathway (PPP) are shown in Fig. 6. No significant perturbations to central carbon metabolism were observed with the combination at 24 h or with either monotherapy at any time. Across the first 4 h of treatment, the polymyxin B/rifampicin combination significantly perturbed phosphoenolpyruvate and pyruvate in glycolysis, and 2-oxoglutarate and acetyl-CoA in TCA cycle (Fig. 6). Several essential genes involved in glycolysis and the TCA cycle, namely PA3416, PA3417, *lpdV*, and *icd*, were also significantly downregulated at 1 h following combination therapy (Fig. 6 and Additional file 1: Table S7). Collectively, these results indicate that glycolysis and the TCA cycle were rapidly inhibited by the combination. Additionally, d-sedoheptulose 7-phosphate, d-ribose 5-phosphate and fructose 6-phosphate, were increased across the first 4 h of the combination treatment, indicating upregulation of pentose phosphate pathway (Fig. 6). Moreover, the results show that the combination caused considerable downregulation (< − 2.0 log_2_FC, FDR ≤ 0.05) of genes associated with aerobic electron transfer, namely PA0521, *cioB*, *cioA* and PA4133 (Table 5).

### The polymyxin B/rifampicin combination significantly altered expression of virulence genes

The polymyxin B/rifampicin combination exclusively reduced the expression of a large number of cellular virulence factors in PAO1 at 1 h (Table 5). Notably, phenazine synthesis (via *phzA1* and *phzA2* operons encoding pyocyanin biosynthesis) was markedly suppressed (< − 2.0 log_2_FC, FDR ≤ 0.05) by the combination treatment at 1 h (Table 5); while polymyxin B alone resulted in the significant upregulation (> 1.0 log_2_FC, FDR ≤ 0.05) of *phzM* and *phzA1* (Table 4). At 1 h the combination also downregulated (< − 1.0 log_2_FC, FDR ≤ 0.05) the genes associated with the Type II secretion system (PA3095–PA3105), Type IV pili (*flp*), Type IV fimbrial biogenesis (PA4551–PA4556), Type VI secretion system (*vgrG6*, *hcpB*), and biofilm formation (PA2231–PA2241). Virulence determinants were predominantly regulated by the quorum sensing (QS) system [55], and the QS-regulatory genes *lasA*, *lasB*, *rhlI*, *rhlR*, *rhlA* and *rhlB* were all downregulated (< − 1.0 log_2_FC, FDR ≤ 0.05) at 1 h by the combination (Table 5). At 1 h, the combination significantly increased the expression of *lasI*, a gene responsible for biosynthesis of QS signal molecule *N*-3-oxo-dodecanoyl homoserine lactone (3OC12-HSL; > 1.0 log_2_FC, FDR ≤ 0.05), as well as the *narK1K2GHJI* operon for nitrate uptake and utilization (> 2.0 log_2_FC, FDR ≤ 0.05). Additionally, the combination significantly downregulated the expression of sigma factor *rpoS*, a general stress response regulator in bacteria (< − 1.0 log_2_FC, FDR ≤ 0.05) [56]. Notably, both polymyxin B alone and the combination significantly upregulated genes (*mexX* and *mexY*) involved in MexXY multidrug efflux transporters (> 1.0 log_2_FC, FDR ≤ 0.05; Tables 4 and 5).

**Table 4:** Differentially expressed genes (> 1.0 log_2_FC and FDR ≤ 0.05) at 1 h induced by polymyxin B monotherapy

Locus tag/gene name	Product description	Expression ratio (log_2_)	Adjusted *p*-value
PA2018 (*mexY*)	RND multidrug efflux transporter	2.82	5.17E−08
PA2019 (*mexX*)	RND multidrug efflux membrane fusion protein	2.89	5.27E−06
PA4611 (*pagL*)	Lipid A 3-*O*-deacylase	1.02	0.00283
PA2354	Transcriptional regulator	2.96	0.00011
PA2356 (*msuD*)	Methanesulfonate sulfonatase	4.24	0.00088
PA2357 (*msuE*)	NADH-dependent FMN reductase	5.55	0.00062
PA4209 (*phzM*)	Phenazine specific methyltransferase	1.34	0.00666
PA4210 (*phzA1*)	Phenazine biosynthesis protein	1.13	0.04192
PA1559	Hypothetical protein	3.06	0.00015
PA1560	Hypothetical protein	2.55	0.00042
PA4774	Hypothetical protein	3.54	0.00066
PA4775	Hypothetical protein	1.44	0.00721
PA4773	Hypothetical protein	3.62	0.00014

**Table 5:** Differentially expressed genes (> 1.0 and < − 1.0 log_2_FC and FDR ≤ 0.05) at 1 h induced by the polymyxin B/rifampicin combination

Gene name/locus tag	Product description	Expression ratio (log_2_)	Adjusted *P*-value
Virulence factor			
*phzA2*, *phzB2*, *phzC2*, *phzD2*, *phzE2*, *phzF2*, *phzG2*, *phzA1*, *phzB1*, *phzC1*, *phzG1*, *phzS*, *phzH*, *phzM*	Phenazine biosynthesis protein	≤ − 2.43 to − 5.01	≤ 0.001
*flp*	Type IV b pilin Flp	− 4.33	3.14E−06
*xcpZ*, *xcpY*, *xcpX*, *xcpW*, *xcpV*, *xcpU*, *xcpT*, *xcpS*, *xcpR*, *xcpP*, *xcpQ*	Type II secretion system protein	< − 1.15 to − 2.03	≤ 0.001
*pslA*, *pslB*, *pslC*, *pslD*, *pslE*, *pslF*, *pslG*, *pslH*, *pslI*, *pslJ*, *pslK*	Biofilm formation protein	< − 1.21 to − 2.09	≤ 0.001
*pilV*, *pilW*, *pilY1*, *pilY2*, *pilE*	Type IV fimbrial biogenesis protein	< − 1.04 to − 1.28	≤ 0.001
PA5265	Hypothetical protein	− 2.84	5.08E−06
*vgrG6*	Type VI secretion system, RhsGE-associated Vgr family subset	− 4.36	5.46E−07
*hcpB*	Secreted protein Hcp	− 4.55	5.20E−07
Antibiotic resistance			
*oprB*	Porin B	− 1.92	0.0005
*oprD*	Porin D	− 1.07	0.006
*mexY*	RND multidrug efflux	2.60	3.92E−08
*mexX*		2.85	9.59E−07
Quorum sensing system			
*lasA*	Protease LasA	− 1.47	5.01E−06
*lasB*	Elastase LasB	− 2.99	3.65E−07
*lasI*	Acyl-homoserine-lactone synthase	1.12	5.35E−06
*rhlI*	Acyl-homoserine-lactone synthase	− 1.33	1.08E−06
*rhlR*	Transcriptional regulator RhlR	− 1.98	7.03E−07
*rhlB*	Rhamnosyltransferase subunit B	− 3.15	3.98E−08
*rhlA*	Rhamnosyltransferase subunit A	− 3.53	3.93E−08
*pqsA*	Anthranilate–CoA ligase	− 1.92	4.37E−07
*pqsB*	Hypothetical protein	− 1.87	7.61E−08
*pqsC*	Hypothetical protein	− 1.76	3.29E−07
*pqsD*	3-Oxoacyl-ACP synthase	− 1.66	4.03E−07
*pqsE*	Thioesterase PqsE	− 1.75	5.62E−07
*pqsH*	2-Heptyl-3-hydroxy-4(1H)-quinolone synthase	− 1.49	7.21E−08
*narI*, *narJ*, *narH*, *narG*	Respiratory nitrate reductase subunit gamma	> 2.16 to 2.64	< 0.05
*narK2*	Nitrite extrusion protein	3.11	< 0.05
*narK1*	Nitrite extrusion protein	3.18	< 0.05
*nirN*	Cytochrome C	− 2.51	2.24E−07
PA0510	Uroporphyrin-III C-methyltransferase	− 2.99	3.93E−08
*nirJ*, *nirH*, *nirG*, *nirL*, *nirD*, *nirF*	Heme d1 biosynthesis protein	< 1.95 to − 3.15	≤ 0.001
*nirC*	Cytochrome c55X	− 2.26	5.33E−06
*nirM*	Cytochrome C-551	− 2.65	1.68E−06
*nirS*	Nitrite reductase	− 2.94	5.35E−06
*norB*	Nitric oxide reductase subunit B	− 2.48	0.00031
*norC*	Nitric oxide reductase subunit B	− 2.42	0.00061
PA0525	Denitrification protein NorD	− 2.30	0.00027
*nosR*	Regulatory protein NosR	− 1.93	1.80E−05
*nosZ*	Nitrous-oxide reductase	− 1.89	5.23E−07
*nosD*	Copper-binding periplasmic protein	− 2.13	1.94E−07
*nosF*	Copper ABC transporter ATP-binding protein	− 2.47	1.24E−05
*nosY*	Membrane protein NosY	− 2.51	3.86E−08
*nosL*	Accessory protein NosL	− 2.03	4.46E−06
Central carbon metabolism/respiration			
PA3928	Hypothetical protein	− 2.86	2.67E−06
*cioB*	Cyanide insensitive terminal oxidase	− 3.20	5.23E−07
*cioA*	Cyanide insensitive terminal oxidase	− 3.43	7.44E−07
PA0521	Cytochrome C oxidase subunit	− 2.24	0.000649
PA4133	cbb3-type cytochrome C oxidase subunit I	− 2.59	1.24E−05
*RpoS*	RNA polymerase sigma factor RpoS	− 1.31	6.61E−06

At 24 h, only 21 DEGs were observed with polymyxin B alone, including the significant upregulation of PA0806 and PA2358 (> 5.0 log_2_FC, FDR ≤ 0.05) and downregulation of *kdpC* (< − 4.0 log_2_FC, FDR ≤ 0.05) (Additional file 1: Table S8). In contrast, 165 DEGs were identified with rifampicin alone, including upregulated genes (> 2.0 log_2_FC, FDR ≤ 0.05) associated with phenazine biosynthesis (Additional file 1: Table S9). Substantially more (*n* = 445) DEGS were observed with the combination, including upregulated genes associated with virulence determinants such as the QS regulatory genes (*rhlR*, *lasI*, *lasA*; > 1.0 log_2_FC, FDR ≤ 0.05), phenazine biosynthesis proteins (*phzH*, *phzA1*, *phzA2*, *phzB2*; > 2.0 log_2_FC, FDR ≤ 0.05), and protease secretion system (*aprD*, *aprE*, *aprF*; > 2.0 log_2_FC, FDR ≤ 0.05) (Additional file 1: Table S10).

## Discussion

Pharmacokinetic/pharmacodynamic (PK/PD) optimization is required for rational antibiotic combination therapy, which can be substantially enhanced by systems investigations on the mechanism of synergistic killing [57]. In the present study, transcriptomic and metabolomic approaches were integrated using strain-specific GSMN modelling to examine the mechanism of synergistic killing by a polymyxin/rifampicin combination against *P. aeruginosa*. PAO1 was employed as it is a major reference strain used for molecular, genomics, biochemical and pharmacological studies on *P. aeruginosa* [1, 58]. GSMN incorporates 4265 biochemical reactions in PAO1 cell and associates the metabolic genes to the corresponding reactions. Most importantly, the GSMN iPAO1 has 1169 and 367 reactions for biosynthesis of lipopolysaccharide and glycerophospholipid, respectively; whereas the corresponding numbers are 49 and 93 in KEGG database [52, 59]. Therefore, we mapped the metabolomic and transcriptomic data to the PAO1 GSMN, which links the changes in gene expression and metabolite level for each reaction and helps to identify the significantly perturbed metabolic reactions and pathways under different antibiotic treatments. Enhanced bacterial killing of PAO1 by the polymyxin B/rifampicin combination occurred rapidly (observable at 0.25 and 1 h) and was driven by polymyxin B (Fig. 1A(I, ii) and Additional file 1: Tables S1 and S2), with minimal or no killing at 24 h by rifampicin (Fig. 1B(iii) and Additional file 2: Table S12). Interestingly, very few significantly changed metabolites were identified at 24 h with either treatment (4 metabolites for rifampicin alone and 14 metabolites for the combination; Fig. 1A(iv) and Additional file 1: Table S4), indicating that the metabolism of PAO1 was not substantially perturbed and that the disorganizing effect of polymyxin B on the outer membrane was minimal.

Notable early (at 0.25 h) changes in membrane-associated lipids with polymyxin B alone or the combination, primarily of glycerophospholipids and fatty acids, were associated with the outer membrane disorganizing action of polymyxin B (Fig. 2) [60]. Similar early changes in membrane-associated lipids have been reported in metabolomic studies of *P. aeruginosa* [61–65] and *A. baumannii* [48, 66, 67] treated with polymyxin (polymyxin B or colistin) alone or combinations. For example, at 1 and 4 h (but not 24 h) fatty acids and lysophospholipids were significantly decreased in a polymyxin-susceptible strain of *P. aeruginosa* PAK treated with 4 mg/L polymyxin B [61]. Significant perturbations to peptidoglycan biosynthesis have also been demonstrated in *P. aeruginosa* with polymyxin B alone and its combinations with tamoxifen [62, 65] and enrofloxacin [63]; and in *A. baumannii* with colistin alone and its combination with doripenem [48], sulbactam [66], and aztreonam [67]. As rifampicin is hydrophobic and does not ordinarily pass through the outer membrane of Gram-negative pathogens [68], significant metabolomic changes were only observed when combined with polymyxin B, the latter likely causing considerable membrane disruption which increased the permeability of the outer membrane to rifampicin, improving access to its target sites within the cytoplasm. Very likely due to the stationary growth, at 24 h the relative abundance of metabolites associated with peptidoglycan and LPS biosynthesis had restored to the control level with any treatment (Figs. 3 and 4).

Polymyxin resistance in *P. aeruginosa* is most commonly due to lipid A modification, a process which is tightly controlled by two-component regulatory systems (TCRs), including PmrAB [69], PhoPQ [70], and ParRS [71]. External stimuli (i.e. cationic antimicrobial peptides such as polymyxin B) and specific mutations in these TCRs result in the phosphorylation of their response regulators (e.g., PmrA, PhoP, ParR) and the expression of the *arnBCADTEF-pmrE* operon which adds 4-aminoarabinose (L-Ara4N) to lipid A phosphates [72]. In the present study, the overexpression of genes of the ParRS-regulated operons (PA1559-PA1560, PA4773-PA4775-*pmrAB* and *arnBCADTEF*) by polymyxin B alone at 1 h (Fig. 4 and Table 4) was consistent with the literature [71, 73]. The upregulation of UDP-glucoronate (Fig. 4), a precursor metabolite for the L-Ara4N synthesis [74], strongly indicates the emergence of polymyxin resistance in PAO1 due to lipid A modification. Furthermore, the significant upregulation of PA4661 (*pagL*) by polymyxin B alone in the present study suggests lipid A deacylation, a process which contributes to polymyxin resistance by decreasing the hydrophobic interaction between the *N*-terminus and positions 6/7 of polymyxins with the fatty acyl chains of lipid A (Table 4) [61]. Interestingly, the activated ParRS system likely induced the MexXY/OprM system [73], with significant upregulation of *mexXY* genes occurring with both polymyxin B alone and the combination (Tables 4 and 5). In addition, the overexpression of two genes associated with phenazine synthesis *phzM* and *phzA1* by polymyxin B alone (Table 4) suggests increased virulence of PAO1 and further in vivo studies are warranted.

Many antibiotics, including rifampicin, cause significant nucleotide perturbations in bacteria [75–77]. Such perturbations have also been observed in *P. aeruginosa* [62, 63, 65] and *A. baumannii* [48, 66, 67] with polymyxins alone or in combinations, and were also evident in the present study with the polymyxin B/rifampicin combination (Fig. 5). Rifampicin treatment significantly increased the levels of nucleotides in *S. aureus*, indicating that bacterial metabolism was arrested [77]. De novo biosynthesis of purine and pyrimidine starts from a common metabolite, phosphoribosyl pyrophosphate (PRPP) from the pentose phosphate pathway [78]. The metabolites of pentose phosphate pathway were increased by the polymyxin B/rifampicin combination (Fig. 6), which indicated the direct correlation with the significant increase of most nucleotides (Fig. 5).

Antibiotic-induced cell death can be due to altered bacterial cellular respiration and central metabolism via carbon flux within the TCA cycle [77, 79, 80]. Several metabolomic and transcriptomic studies have demonstrated that antibiotics (i.e. ampicillin, kanamycin, gentamicin, rifampicin and norfloxacin) significantly increase the rate of cellular metabolism in treated bacteria [77, 81]. Polymyxins can differentially alter the levels of metabolites associated with central carbon metabolism in *P. aeruginosa* [61–63, 65] and *A. baumannii* [48, 66, 67]*.* In the present study, both metabolomic and transcriptomic data consistently showed that only the polymyxin B/rifampicin combination significantly perturbed the central carbon metabolic pathways including glycolysis, TCA cycle and PPP in *P. aeruginosa* PAO1 (Fig. 6). The combination inhibited the conversion of phosphoenolpyruvate (levels of which were increased) to pyruvate (levels decreased) by downregulating several associated genes (e.g. PA3416, PA3417).

RpoS is a sigma factor and a general stress response regulator in bacteria; and is positively regulated as a counter-measure to conditions that include osmotic and oxidative stress [82]. In the present study, *rpoS* was significantly downregulated at 1 h by the combination (Table 5). Significant suppression of *rpoB* by rifampicin alone and the combination at 24 h (Additional file 2: Table S12) indicates the reduced expression of the β subunit of RNA polymerase in *P. aeruginosa* PAO1, thereby reducing the binding with rifampicin and survival. Moreover, at 24 h the differentially upregulated *sodM* (encoding superoxide dismutase) suggests that rifampicin alone and the synergistic combination caused oxidative stress in PAO1 that was mainly driven by rifampicin (Additional file 2: Table S12).

Another major finding of the present study is that at 1 h the polymyxin B/rifampicin combination significantly downregulated the expression of genes involved with quorum sensing and virulence (e.g., phenazine biosynthesis genes) (Table 5). The two quorum sensing systems *las* and *rhl* in *P. aeruginosa* are potential antimicrobial targets due to their involvement in pathogenicity [55]. Chorismate serves as a precursor for aromatic amino acid and phenazine biosynthesis at the branch point of the shikimic acid pathway [83]. The polymyxin B/rifampicin combination significantly depleted intracellular phenylpyruvate and l-tryptophan (Table 2 and Additional file 1: Table S2) which are intermediate metabolites of aromatic amino acid biosynthesis, indicating that chorismate metabolism was significantly downregulated. This observation aligns well with suppression of the phenazine biosynthesis operons (i.e., *phzA1* and *phzA2*) by the combination (Table 5). Given the important role of quorum sensing in the virulence of *P. aeruginosa* [55], as well as the additional suppression of other virulence factors by the combination, clinical use of the polymyxin B/rifampicin combination has the potential to not only increase bacterial killing but also decrease pathogenesis. Further studies on the effect of this combination on pathogenesis are warranted.

Additionally, we identified perturbations to the denitrification genes at 1 h by the combination, including the enhanced expression of *narK1K2GHJI* operon (nitrate uptake and utilization) and decreased expression of *nirSMCFDLGHJEN* operon (nitrite reduction and heme biosynthesis, Table 5). It was demonstrated that the transcription of *nar* operon requires binding of the activated 3OC12-HSL receptor LasR to its promoter region [84]. Here, we discovered that *lasI*, the critical gene for 3OC12-HSL biosynthesis, was also upregulated at 1 h by the combination. This is very likely because 3OC12-HSL activated the LasI cognate receptor LasR, which in turn positively regulated the expression of *nar* operon as previously reported [84]. Since the medium was not supplemented with nitrate or nitrite in the present study, it is unlikely that *P. aeruginosa* PAO1 employed denitrification as a major energy source to sustain cellular activities. The mechanism of differential expression of denitrification genes remains unclear and warrants further investigation.

## Conclusions

To the best of our knowledge, this study is the first to apply an integrated systems pharmacology approach to investigate synergistic killing of *P. aeruginosa* by polymyxin/rifampicin combination. Using our PAO1 GSMN with a detailed representation of lipopolysaccharide and glycerophospholipid biosynthesis, integrative analysis of transcriptomic and metabolomic results substantially enhanced the identification of significantly perturbed key metabolic pathways by the combination, in particular membrane lipid and peptidoglycan synthesis, nucleotide and amino acid metabolism. Interestingly, the combination also substantially impacted quorum sensing regulation and virulence factors. Better understanding of the complex bacterial metabolic and transcriptomic responses to antibiotic combinations will assist in the optimization of their use in patients.

## Supplementary Information

**Additional file 1: Table S1.** Significantly changed metabolites at 15 min. **Table S2.** Significantly changed metabolites at 1 h. **Table S3.** Significantly changed metabolites at 4 h. **Table S4.** Significantly changed metabolites at 24 h. **Table S5.** DEGs induced by polymyxin B at 1 h. **Table S6.** DEGs induced by rifampicin B at 1 h. **Table S7.** DEGs induced by the combination of polymyxin B and rifampicin at 1 h. **Table S8.** DEGs induced by polymyxin B at 24 h. **Table S9.** DEGs induced by rifampicin at 24 h. **Table S10.** DEGs induced by the combination of polymyxin B and rifampicin at 24 h.

**Additional file 2: Figure S1.** Time-kill kinetics of polymyxin B (PolyB; 1 mg/L), rifampicin (Rif; 2 mg/L), and their combination against *P. aeruginosa* PAO1 at starting 600 nm (OD_600_) of ~ 0.5 (~ 10^8^ CFU/mL). **Figure S2.** GO enrichment analysis of the significantly changed genes with the polymyxin B/rifampicin combination at (**A**) 1 h and (**B**) 24 h: (i) downregulation and (ii) upregulation of various biological processes. **Figure S3.** Overview of metabolic pathways of *P. aeruginosa* PAO1 affected by the polymyxin B/rifampicin combination at (**A**) 1 h and (**B**) 24 h. Blue edges represent the significantly changed enzymatic reactions and red nodes represent the significantly changed metabolites. Significant metabolites and DEGs (including both up- and down-regulated) were identified with > 1.0 and < − 1.0 log_2_FC and FDR ≤ 0.05. **Table S11.** Common differentially expressed genes (> 1.0 and < − 1.0 log_2_FC and FDR ≤ 0.05) induced by polymyxin B (PB) alone and the polymyxin B/rifampicin combination (COMBO) at 1 h and 24 h. **Table S12.** Common differentially expressed genes (> 1.0 and < − 1.0 log_2_FC and FDR ≤ 0.05) induced by rifampicin (RIF) alone and the polymyxin B/rifampicin combination (COMBO) at 24 h.

## Data Availability

All the data that support the findings of this study are available in the article and Additional files.

## Abbreviations

MDR: Multidrug-resistant
CDC: Centers for Disease Control and Prevention
LPS: Lipopolysaccharide
PK/PD: Pharmacokinetics/pharmacodynamics
MIC: Minimum inhibitory concentration
CAMHB: Cation-adjusted Mueller–Hinton broth
CLSI: Clinical and Laboratory Standards Institute
DMSO: Dimethyl sulfoxide
DEG: Differential gene expression
FDR: False discovery rate
REVIGO: Reduce and Visualize Gene ontology
CMW: Chloroform:methanol:water
ESI: Electro-spray ionization
PCA: Principal component analysis
GSMN: Genome-scale metabolic network
GO: Gene ontology
PE: Phosphatidylethanolamine
PG: Phosphatidylglycerol
TCA: Tricarboxylic acid
PPP: Pentose phosphate pathway
QS: Quorum sensing
TCR: Two-component regulatory system
PRPP: Phosphoribosyl pyrophosphate
