# Supplementary material for: Integrated metabolomic and transcriptomic analyses of the synergistic effect of polymyxin–rifampicin combination against Pseudomonas aeruginosa
Source: J Biomed Sci. 2022 Oct 30;29:89. doi: 10.1186/s12929-022-00874-3 (PMC9618192; doi:10.1186/s12929-022-00874-3)
Supplement: Supplementary file 2 — Additional file 2: Figure S1. Time-kill kinetics of polymyxin B (PolyB; 1 mg/L), rifampicin (Rif; 2 mg/L), and their combination against P. aeruginosa PAO1 at starting 600 nm (OD600) of ~ 0.5 (~ 108 CFU/mL). Figure S2. GO enrichment analysis of the significantly changed genes with the polymyxin B/rifampicin combination at (A) 1 h and (B) 24 h: (i) downregulation and (ii) upregulation of various biological processes. Figure S3. Overview of metabolic pathways of P. aeruginosa PAO1 affected by the polymyxin B/rifampicin combination at (A) 1 h and (B) 24 h. Blue edges represent the significantly changed enzymatic reactions and red nodes represent the significantly changed metabolites. Significant metabolites and DEGs (including both up- and down-regulated) were identified with > 1.0 and < − 1.0 log2FC and FDR ≤ 0.05. Table S11. Common differentially expressed genes (> 1.0 and < − 1.0 log2FC and FDR ≤ 0.05) induced by polymyxin B (PB) alone and the polymyxin B/rifampicin combination (COMBO) at 1 h and 24 h. Table S12. Common differentially expressed genes (> 1.0 and < − 1.0 log2FC and FDR ≤ 0.05) induced by rifampicin (RIF) alone and the polymyxin B/rifampicin combination (COMBO) at 24 h. [file 12929_2022_874_MOESM2_ESM.docx]

**Additional file 2**


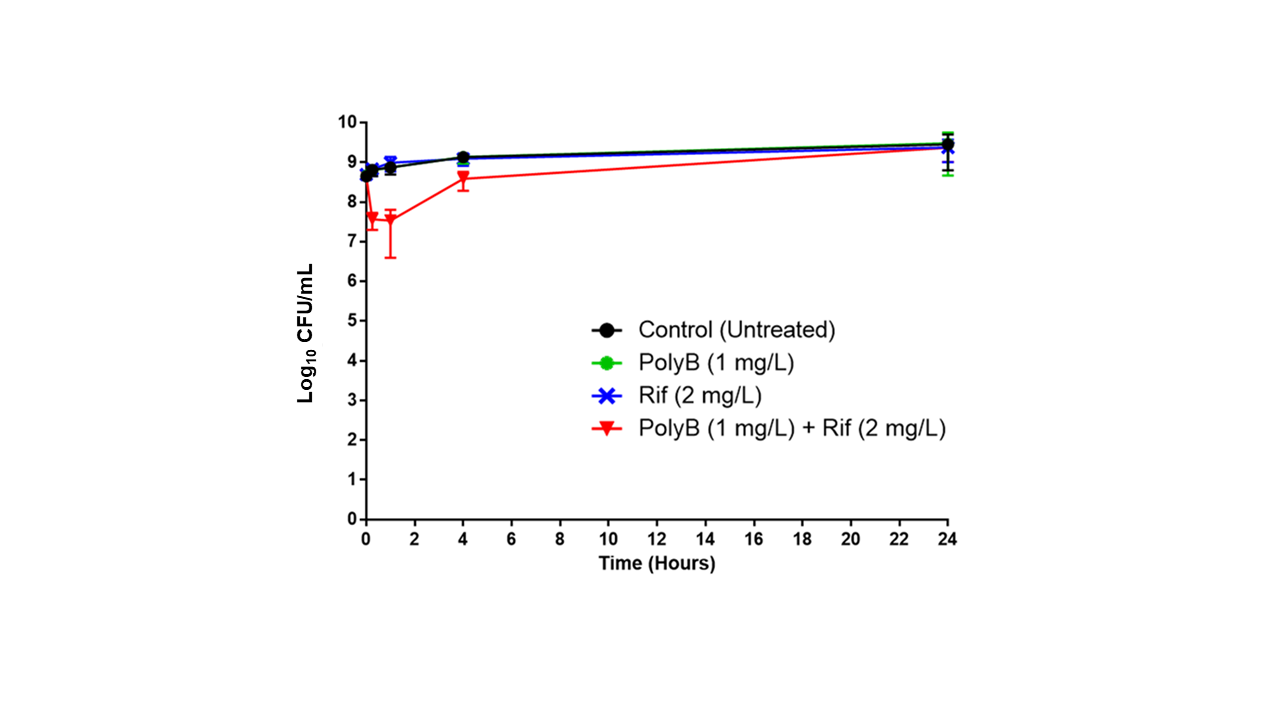


**Fig. S1.** Time-kill kinetics of polymyxin B (PolyB; 1 mg/L), rifampicin (Rif; 2 mg/L), and their combination against *P. aeruginosa* PAO1 at starting 600 nm (OD_600_) of ~0.5 (~10^8^ CFU/mL).


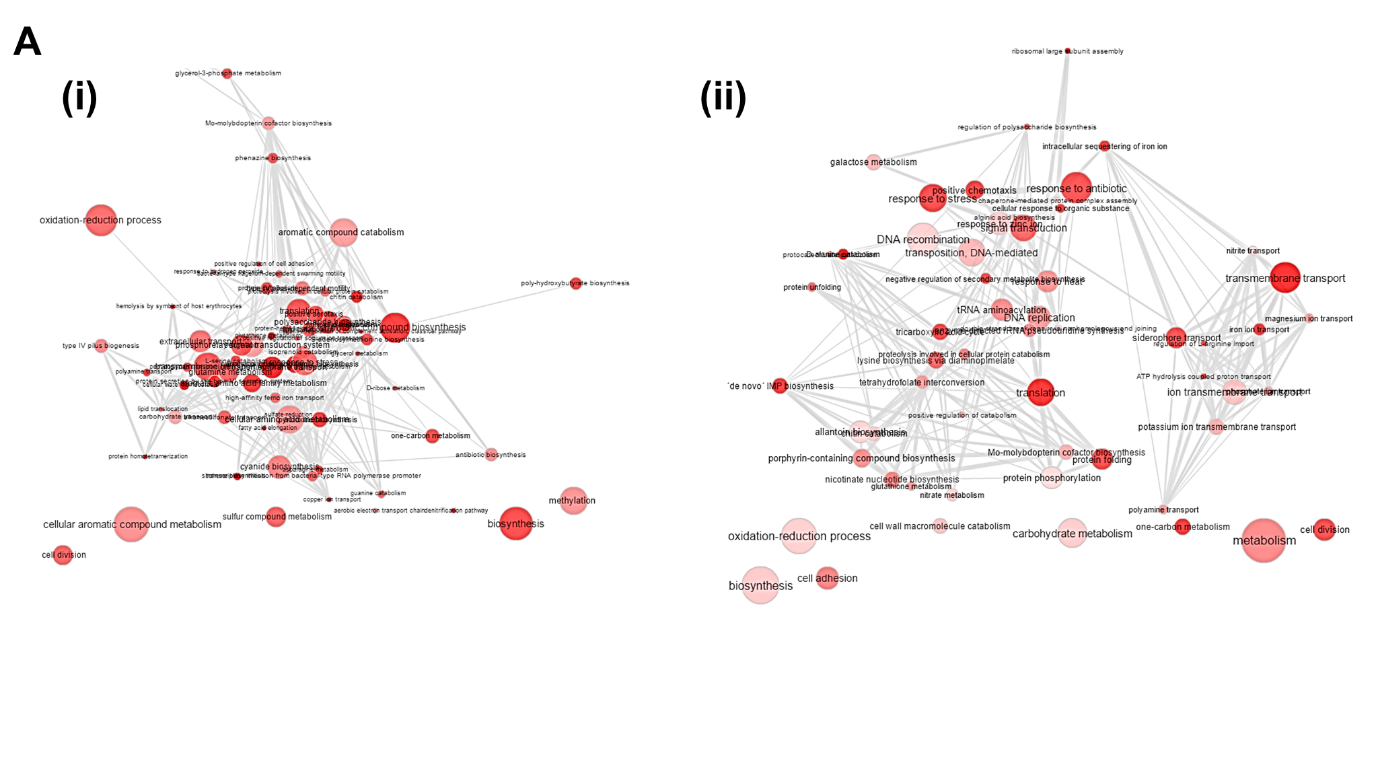


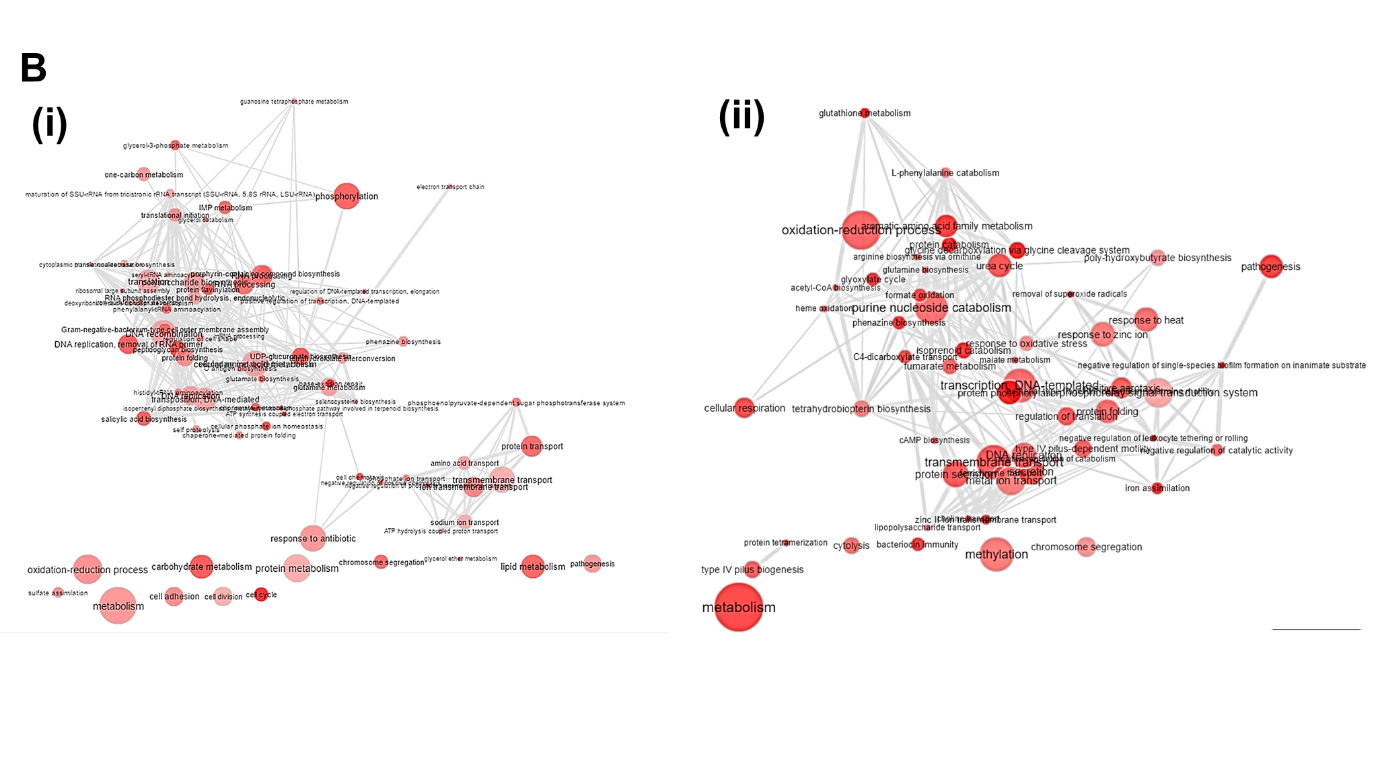


**Fig. S2.** GO enrichment analysis of the significantly changed genes with the polymyxin B/rifampicin combination at (A) 1 h and (B) 24 h: (i) downregulation and (ii) upregulation of various biological processes.


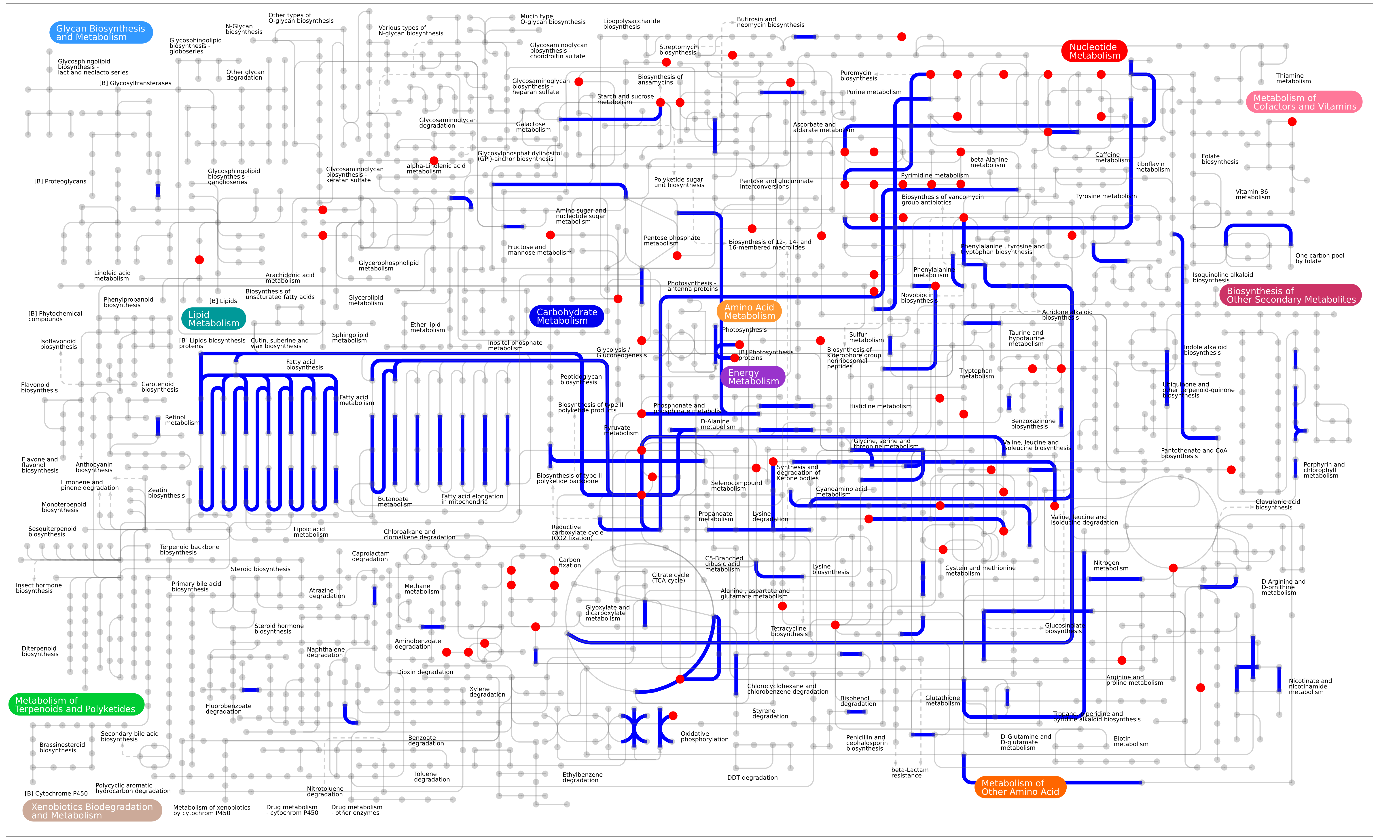


**B**

**A**


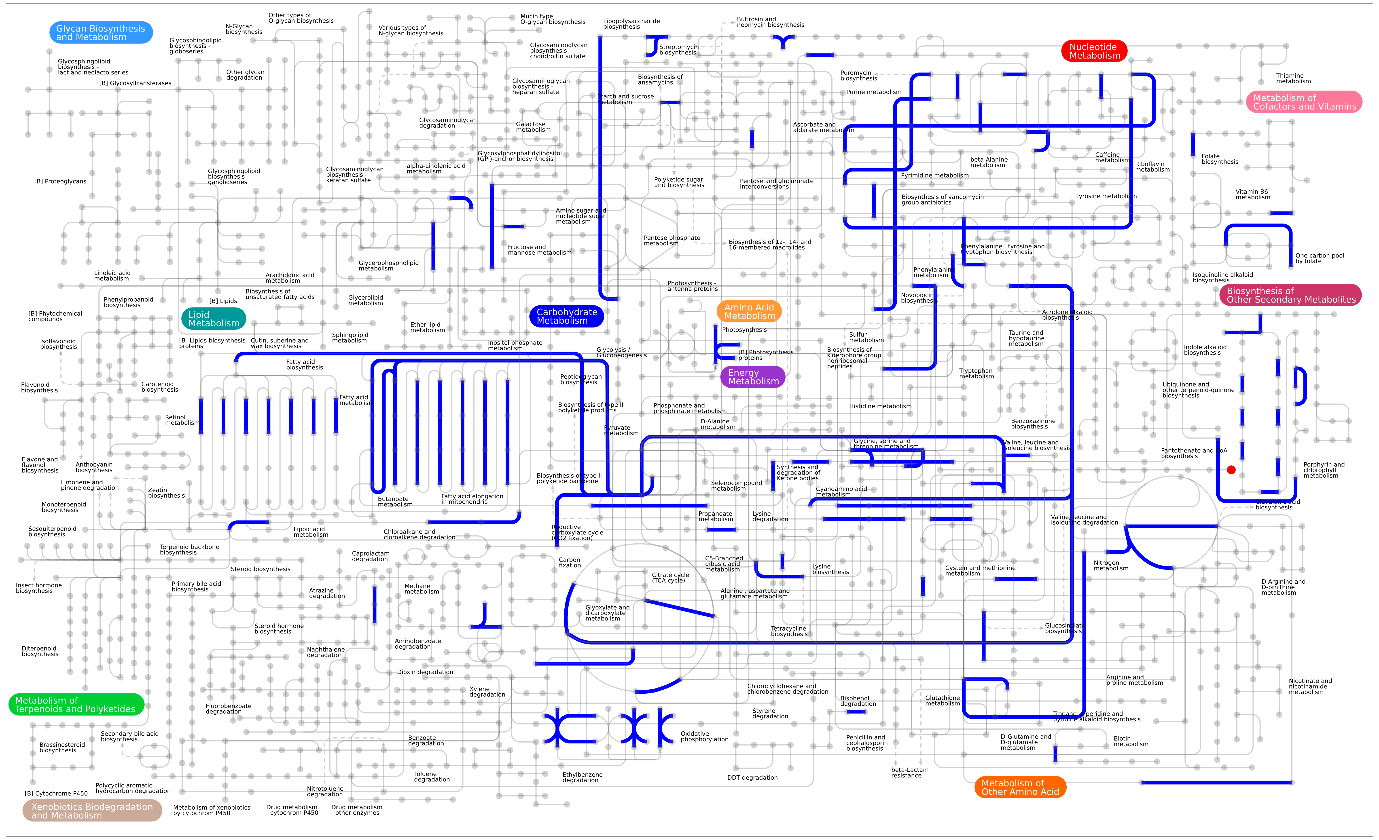


**Fig. S3.** Overview of metabolic pathways of *P. aeruginosa* PAO1 affected by the polymyxin B/rifampicin combination at **(A)** 1 h and **(B)** 24 h. Blue edges represent the significantly changed enzymatic reactions and red nodes represent the significantly changed metabolites. Significant metabolites and DEGs (including both up- and down-regulated) were identified with >1.0 and <-1.0 log_2_FC and FDR ≤ 0.05.

**Table S11** Common differentially expressed genes (>1.0 and <-1.0 log_2_FC and FDR ≤ 0.05) induced by polymyxin B (PB) alone and the polymyxin B/rifampicin combination (COMBO) at 1 h and 24 h.

| Locus tag | Product description | Expression ratio (log_2_) | | | |
| --- | --- | --- | --- | --- | --- |
|  |  | PB  (1 h) | COMBO  (1 h) | PB  (24 h) | COMBO  (24 h) |
| PA1797 | Hypothetical protein | 5.20 | 3.40 | 3.05 | 1.82 |
| PA0806 | Hypothetical protein | 5.11 | 2.89 | 5.50 | 3.03 |
| PA2358 | Hypothetical protein | 5.72 | 3.81 | 5.00 | 3.47 |

**Table S12** Common differentially expressed genes (>1.0 and <-1.0 log_2_FC and FDR ≤ 0.05) induced by rifampicin (RIF) alone and the polymyxin B/rifampicin combination (COMBO) at 24 h.

| Gene name/  Locus tag | Product description | Expression ratio (log_2_) | |
| --- | --- | --- | --- |
|  |  | **RIF** | **COMBO** |
| *phzH* | phenazine-modifying protein | 2.25 | 2.62 |
| PA0133 | HTH-type transcriptional activator BauR | 1.42 | 1.23 |
| *nuh* | nonspecific ribonucleoside hydrolase | 1.44 | 1.47 |
| *aer2* | aerotaxis transducer Aer2 | 2.52 | 2.91 |
| PA0187 | hypothetical protein | 2.30 | 2.66 |
| PA0188 | hypothetical protein | 1.88 | 1.98 |
| *fiuA* | ferrichrome receptor FiuA | 2.01 | 2.27 |
| PA0493 | acetyl-CoA carboxylase biotin carboxyl carrier protein subunit | 1.64 | 1.40 |
| PA0494 | acetyl-CoA carboxylase biotin carboxylase subunit | 1.70 | 1.36 |
| PA0495 | hypothetical protein | 1.81 | 1.36 |
| PA0572 | hypothetical protein | 1.44 | 1.85 |
| PA0586 | hypothetical protein | 1.89 | 1.82 |
| PA0587 | hypothetical protein | 1.07 | 1.44 |
| *hemO* | heme oxygenase | 2.06 | 2.43 |
| *vreA* | hypothetical protein | 2.05 | 2.38 |
| PA0716 | hypothetical protein | 1.05 | 1.15 |
| *recO* | DNA repair protein RecO | -2.10 | -2.29 |
| *hpd* | 4-hydroxyphenylpyruvate dioxygenase | 3.38 | 3.26 |
| *phhB* | pterin-4-alpha-carbinolamine dehydratase | 1.63 | 1.95 |
| PA0894 | hypothetical protein | -1.67 | -1.91 |
| PA0984 | colicin immunity protein | 2.00 | 2.16 |
| *pyoS5* | pyocin S5 | 1.43 | 1.69 |
| *braE* | branched-chain amino acid ABC transporter permease BraE | 1.83 | 1.19 |
| PA1131 | major facilitator superfamily transporter | -1.12 | -1.24 |
| *aprE* | alkaline protease secretion protein AprE | 3.00 | 3.76 |
| PA1274 | 5,6-dimethylbenzimidazole synthase | -1.66 | -2.69 |
| *cobD* | cobalamin biosynthesis protein CobD | -2.04 | -2.13 |
| *cobC* | threonine-phosphate decarboxylase | -2.49 | -2.49 |
| PA1302 | heme utilization protein | 2.07 | 2.48 |
| PA1351 | ECF subfamily sigma-70 factor | 1.30 | 1.15 |
| *lasI* | acyl-homoserine-lactone synthase | 1.38 | 1.11 |
| *sucB* | 2-oxoglutarate dehydrogenase complex dihydrolipoyllysine-residue succinyltransferase | -1.30 | -1.24 |
| *kdpB* | potassium-transporting ATPase subunit B | -2.98 | -2.52 |
| PA1833 | Oxidoreductase | 1.11 | 1.32 |
| *phza2* | phenazine biosynthesis protein PhzA | 1.80 | 2.50 |
| *maiA* | maleylacetoacetate isomerase | 2.49 | 2.98 |
| *fahA* | Fumarylacetoacetase | 3.33 | 3.99 |
| *liuC* | gamma-carboxygeranoyl-CoA hydratase | 3.06 | 2.56 |
| *liuB* | methylcrotonyl-CoA carboxylase subunit beta | 2.12 | 1.76 |
| *liuA* | isovaleryl-CoA dehydrogenase | 2.28 | 1.76 |
| PA2033 | hypothetical protein | 1.99 | 2.35 |
| PA2034 | hypothetical protein | 1.68 | 1.93 |
| PA2226 | hypothetical protein | 1.09 | 1.44 |
| *vqsM* | HTH-type transcriptional regulator VqsM | 1.27 | 1.38 |
| *bkdB* | branched-chain alpha-keto acid dehydrogenase complex lipoamide acyltransferase | 1.01 | 1.35 |
| *ansA* | L-asparaginase I | -1.49 | -1.12 |
| PA2381 | hypothetical protein | 1.28 | 1.26 |
| PA2407 | adhesion protein | 2.54 | 2.81 |
| *gcvP2* | glycine dehydrogenase | 2.05 | 2.55 |
| PA2465 | hypothetical protein | 2.51 | 2.13 |
| PA2540 | hypothetical protein | -1.18 | -1.37 |
| PA2552 | acyl-CoA dehydrogenase | 1.70 | 1.29 |
| PA2553 | acyl-CoA thiolase | 1.93 | 1.74 |
| PA2554 | short-chain dehydrogenase | 1.99 | 1.52 |
| PA2561 | methyl-accepting chemotaxis protein CtpH | 1.04 | 1.72 |
| PA2838 | transcriptional regulator | -1.74 | -1.49 |
| PA2902 | hypothetical protein | -1.06 | -1.13 |
| *cobJ* | precorrin-3 methylase CobJ | -1.31 | -1.77 |
| PA2914 | ABC transporter permease | -1.77 | -1.57 |
| *nqrF* | Na(+)-translocating NADH-quinone reductase subunit F | -1.75 | -2.19 |
| *rmf* | ribosome modulation factor | 1.36 | 1.86 |
| *xcpT* | type II secretion system protein G | 1.25 | 1.13 |
| PA3122 | transcriptional regulator | 1.18 | 1.35 |
| PA3174 | transcriptional regulator | 1.12 | 1.07 |
| *glpD* | glycerol-3-phosphate dehydrogenase | -1.21 | -2.05 |
| *lpxB* | lipid-A-disaccharide synthase | -1.14 | -1.31 |
| *cdsA* | phosphatidate cytidylyltransferase | -1.71 | -2.30 |
| *wspE* | chemotaxis sensor/effector fusion protein | -1.04 | -1.24 |
| PA3711 | transcriptional regulator | 1.13 | 1.15 |
| PA3723 | FMN oxidoreductase | 1.23 | 1.31 |
| *exoS* | exoenzyme S | 1.45 | 1.63 |
| *rocR* | DNA-binding response regulator RocR | 1.67 | 1.32 |
| *sltB1* | soluble lytic transglycosylase B | -1.09 | -1.12 |
| *rodA* | rod shape-determining protein | -1.64 | -1.79 |
| PA4048 | hypothetical protein | -1.49 | -1.85 |
| PA4090 | hypothetical protein | 1.81 | 1.51 |
| *phza1* | phenazine biosynthesis protein | 2.60 | 2.06 |
| *pchD* | 2,3-dihydroxybenzoate-AMP ligase | 2.59 | 3.20 |
| *rpsN* | 30S ribosomal protein S14 | -2.83 | -2.50 |
| *rpsQ* | 30S ribosomal protein S17 | -2.56 | -3.05 |
| *rpmC* | 50S ribosomal protein L29 | -2.26 | -3.02 |
| *rpoB* | DNA-directed RNA polymerase subunit beta | -1.47 | -1.88 |
| *mvaT* | transcriptional regulator MvaT | 1.05 | 1.13 |
| *icmP* | insulin-cleaving metalloproteinase outer membrane protein | 1.96 | 2.07 |
| PA4467 | hypothetical protein | 2.90 | 3.48 |
| *sodM* | superoxide dismutase | 3.03 | 3.55 |
| PA4469 | hypothetical protein | 3.06 | 3.74 |
| *fumC1* | fumarate hydratase | 2.44 | 3.05 |
| PA4476 | hypothetical protein | -1.23 | -1.30 |
| PA4497 | ABC transporter | 1.78 | 1.56 |
| *opdP* | glycine-glutamate dipeptide porin OpdP | 1.80 | 1.73 |
| *lytB* | 4-hydroxy-3-methylbut-2-enyl diphosphate reductase | -1.34 | -1.63 |
| PA4570 | hypothetical protein | 1.79 | 2.07 |
| *mscL* | large-conductance mechanosensitive channel | 1.06 | 1.23 |
| *murI* | glutamate racemase | -1.27 | -2.23 |
| PA4680 | hypothetical protein | 2.40 | 2.31 |
| PA4706 | hemin importer ATP-binding subunit | 1.53 | 1.35 |
| *pnp* | polynucleotide phosphorylase | -1.15 | -1.29 |
| *rpsO* | 30S ribosomal protein S15 | -1.10 | -1.24 |
| *truB* | tRNA pseudouridine synthase B | -1.35 | -1.48 |
| PA4754 | hypothetical protein | -1.16 | -1.11 |
| *greA* | transcription elongation factor GreA | -1.26 | -1.48 |
| *fdnI* | nitrate-inducible formate dehydrogenase subunit gamma | 2.20 | 1.39 |
| *fdnH* | nitrate-inducible formate dehydrogenase subunit beta | 2.41 | 2.13 |
| *retS* | sensor histidine kinase MifS | -1.92 | -2.35 |
| PA4889 | Oxidoreductase | -1.16 | -1.64 |
| *parC* | DNA topoisomerase IV subunit A | -1.11 | -1.47 |
| PA5008 | hypothetical protein | -1.76 | -2.32 |
| *pilQ* | type IV fimbrial biogenesis outer membrane protein PilQ | 1.21 | 1.19 |
| PA5061 | hypothetical protein | 1.22 | 1.19 |
| *glnA* | glutamine synthetase | 1.39 | 1.60 |
| *dctP* | C4-dicarboxylate-binding protein | 1.51 | 1.37 |
| *dctQ* | dicarboxylate transporter | 2.43 | 2.10 |
| *dctM* | C4-dicarboxylate transporter | 2.37 | 1.99 |
| PA5230 | ABC transporter permease | 1.65 | 2.23 |
| PA5312 | aldehyde dehydrogenase | 1.48 | 1.06 |
| *pstB* | phosphate ABC transporter ATP-binding protein | -1.82 | -2.04 |
| *betA* | choline dehydrogenase | -1.20 | -2.69 |
| *betI* | BetI family transcriptional regulator | -1.17 | -1.98 |
| *wzm* | LPS efflux transporter membrane protein | 1.75 | 1.25 |
| *wbpW* | phosphomannose isomerase/mannose-1-phosphate guanylyl transferase | 1.81 | 1.73 |
| *atpC* | ATP synthase subunit epsilon | -2.82 | -2.53 |
| *atpD* | ATP synthase subunit beta | -2.97 | -2.94 |
